# Optogenetic manipulation and photoacoustic imaging using a near-infrared transgenic mouse model

**DOI:** 10.1038/s41467-022-30547-6

**Published:** 2022-05-19

**Authors:** Ludmila A. Kasatkina, Chenshuo Ma, Mikhail E. Matlashov, Tri Vu, Mucong Li, Andrii A. Kaberniuk, Junjie Yao, Vladislav V. Verkhusha

**Affiliations:** 1 Department of Genetics and Gruss-Lipper Biophotonics Center, Albert Einstein College of Medicine, Bronx, NY 10461 USA; 2 Department of Biomedical Engineering, Duke University, Durham, NC 27708 USA; 3 Medicum, Faculty of Medicine, University of Helsinki, Helsinki, 00290 Finland

**Keywords:** Optogenetics, Imaging, Mouse

## Abstract

Optogenetic manipulation and optical imaging in the near-infrared range allow non-invasive light-control and readout of cellular and organismal processes in deep tissues in vivo. Here, we exploit the advantages of *Rhodopseudomonas palustris* BphP1 bacterial phytochrome, which incorporates biliverdin chromophore and reversibly photoswitches between the ground (740–800 nm) and activated (620–680 nm) states, to generate a *loxP-BphP1* transgenic mouse model. The mouse enables Cre-dependent temporal and spatial targeting of BphP1 expression in vivo. We validate the optogenetic performance of endogenous BphP1, which in the activated state binds its engineered protein partner QPAS1, to trigger gene transcription in primary cells and living mice. We demonstrate photoacoustic tomography of BphP1 expression in different organs, developing embryos, virus-infected tissues and regenerating livers, with the centimeter penetration depth. The transgenic mouse model provides opportunities for both near-infrared optogenetics and photoacoustic imaging in vivo and serves as a source of primary cells and tissues with genomically encoded BphP1.

## Abstract (web summary)

Optogenetic tools can be used as in vivo imaging probes. Here the authors generate a loxP-BphP1 transgenic mouse to enable Cre-dependent temporal and spatial targeting of BphP1 expression in vivo; they show photoacoustic tomography of BphP1 expression in developing embryos and regenerating livers.

## Introduction

Optogenetic manipulation of cellular processes in their native environment and deep-tissue imaging in vivo are essential for non-invasive studies of various biological problems. For this purpose, genetically encoded optogenetic tools (OTs)^1,2^ and imaging probes^3,4^ should operate in the near-infrared (NIR) spectral range. NIR light allows deep-tissue penetration due to low tissue absorbance, low autofluorescence and reduced light-scattering^5^. Several NIR OTs, NIR fluorescent proteins (FPs) including iRFPs^6,7^, and NIR photoacoustic (PA) imaging probes have been designed from soluble bacterial phytochrome photoreceptors (BphPs)^7,8^. Being natural molecular photosensors in bacteria, fungi and plants, phytochromes serve as mediators in a variety of light-dependent physiological processes. BphPs exhibit reversible photoswitching from the biologically inactive ground state to the functional active state. An important advantage of BphPs and BphP-derived constructs over probes based on plant or cyanobacterial photoreceptors is that their chromophore biliverdin IXa is readily available as the product of heme catabolism in eukaryotes including mammals. BphPs with biliverdin covalently bound in its chromophore-binding pocket photoconverts between the Pr and Pfr conformational states (Supplementary Fig. 1).

In vivo suitability and non-toxicity of BphP-derived constructs have been validated in transgenic animals expressing different NIR FPs, including *Drosophila*^9,10^, mouse^11–14^ and rat^15^. One type of BphP-based OTs relies on a BphP from *Rhodopseudomonas palustris*, called RpBphP1 (below shortened to BphP1), which together with a transcriptional regulator PpsR2^16^ comprises a NIR light-sensitive system to control the expression of photosynthetic apparatus in bacteria^17^. Being in the ground so-called Pfr state, BphP1 undergoes reversible NIR light-induced transition to the Pr state and, consequently, binds the PpsR2 protein partner, mediating transcription regulation^18,19^. Since the applications of the PpsR2 are limited by its large size, multidomain structure and oligomerization, we recently engineered a single-domain BphP1 binding partner, termed QPAS1, which is three-fold smaller and lacks oligomerization while efficiently interacts with BphP1^20,21^. This latter BphP1-QPAS1 OT has a high potential as modification can be introduced to the system like membrane-tethering, inclusion of catalytic domains or blue-light sensitive nuclear-localizing domains, enabling not only gene transcription regulation, but also controllable protein targeting, enzymatic activity and spectral multiplexing with blue-light OTs^20–22^.

In living organisms, the concentration of endogenous biliverdin, as a chromophore for BphP1 and iRFPs, varies among tissues. Taking into account that BphP1 apoform (without bound biliverdin chromophore) may interact with QPAS1, exogenous overexpression of BphP1 together with low cellular (endogenous) biliverdin level results in a notable amount of BphP1 apoform and, consequently, in a high background of OT action in darkness. This issue has been resolved by the development of cell lines that stably express BphP1 at low levels, because low BphP1 concentration favors its complete assembling with endogenous biliverdin into a holoform^19,22^. By analogy, generation of a transgenic mouse encoding two copies of the BphP1 gene per diploid cell can help to alleviate this problem for in vivo BphP1-QPAS1 applications.

Another advantage of the BphP is its use as PA molecular probe at depths far beyond that attainable by other optical imaging technologies^23–25^. PA tomography (PAT) is a hybrid imaging modality that detects light-induced ultrasound waves and has intrinsic sensitivity to the optical absorption contrast agents. PAT takes full advantage of the strong NIR light absorption of BphP and the weak acoustic attenuations of the biological tissue and can achieve high spatial resolution imaging at centimeter depth. Moreover, the reversible photoswitching capability of BphP has enabled the differential detection in PAT that can suppress the non-switching signals from blood hemoglobin and improve the molecular detection sensitivity by three orders of magnitude^23,24^.

By allowing the live imaging of embryogenesis, tissue regeneration, and pathology progression, fluorescent-reporter mouse strains are now among the most commonly used large-fragment knock-in mouse models^12^. However, these models are limited to fluorescence imaging with shallow imaging depths. While PAT has been increasingly used for deep-tissue studies in animal models, to date there have been no transgenic mouse models specifically expressing reversibly photoswitchable PA probes. Given the superior optical properties of BphP1 as a probe for differential PAT, a transgenic BphP1 mouse model is needed for broadening PAT applications in deep-tissue in vivo studies.

Here, we exploit the advantages of BphP1 having strong NIR light absorption (at 740–800 nm light for its Pfr ground state) and reversible photoswitching back from activated Pr state (at 620–680 nm), to generate a transgenic mouse model that can be used for both, NIR optogenetic manipulation and deep-tissue PAT. We generated a *loxP-BphP1* knock-in mouse that bears the *BphP1-mCherry-TetR* transgene construct under the control of *loxP* sites to allow Cre recombinase-dependent temporal and spatial targeting of its expression^26^. We validated the optogenetic performance of endogenous BphP1-mCherry-TetR protein to activate gene transcription in primary cells and living mice using NIR light. Moreover, Cre-mediated expression of the same BphP1 construct enabled deep-tissue PA imaging of different internal organs, developing embryos, virus-targeted tissues, and regenerating livers, with high detection sensitivity.

## Results

### Generation of loxP-BphP1 knock-in mouse and transgene characterization

**Fig. 1:**
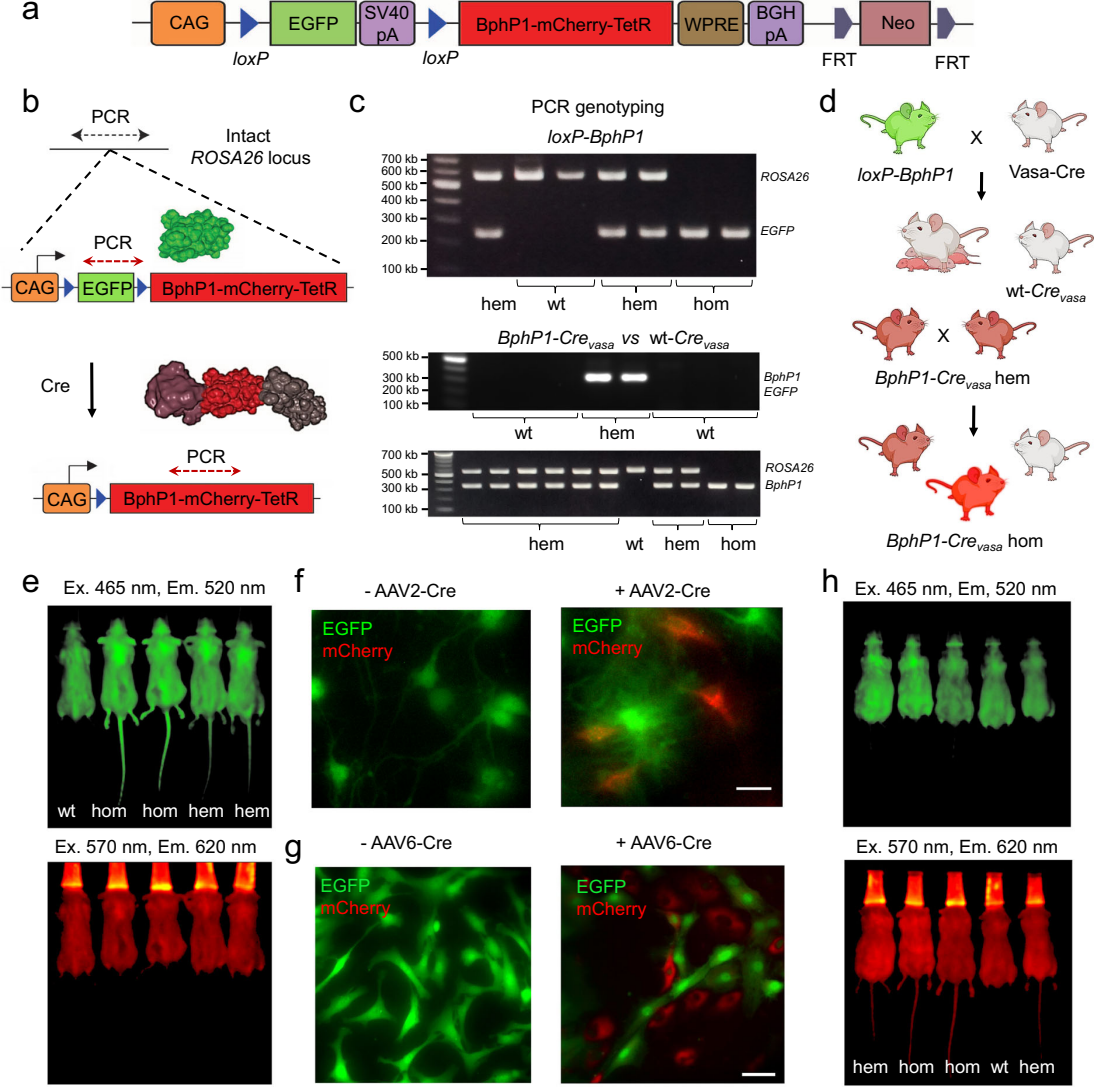
Generation of *loxP-BphP1-mCherry-TetR* knock-in mice and Cre-dependent BphP1 expression. **a** Transgene elements: CAG, CAG promoter; *loxP*, locus of X-over P1, Cre-recombinase recognition site; SV40pA, simian virus 40 polyadenylation signal; TetR, tetracycline repressor protein; WPRE, Woodchuck Hepatitis Virus (WHP) Posttranscriptional Regulatory Element; BGHpA, bovine growth hormone polyadenylation signal; FRT, flippase recognition target site; Neo, neomycin resistance cassette. **b** Targeting of *BphP1* gene into *ROSA26* locus and Cre-dependent expression of BphP1-mCherry-TetR protein. **c** PCR genotyping of *loxP*-*BphP1* and *BphP1-Cre*_*vasa*_ mice using primers annealing to intact *ROSA26* locus (5′-AGCACTTGCTCTCCCAAAGTC, 5′-TGCTTACATAGTCTAACTCGCGAC, product size 564 bp), transgene *EGFP* sequence (5′-GGCAGAGGATCGTTTCGCGG, 5′-GAAGCACTGCACGCCGTAGG, product size 240 bp) and *BphP1* sequence (5′-GAGCGCGTCCACACCGTTAC, 5′-GGCAACATGTCGCGGAACGA, product size 341 bp). Genotypes indicated: hom, homozygote; hem, hemizygote; and wt, wild-type. **d** Hemizygous *loxP-BphP1* males were crossed with Cre-driver (Vasa-Cre) strain females of ubiquitously active Cre, and subsequent generations of homozygous *BphP1-Cre*_*vasa*_ mice were obtained. **e** In vivo imaging of the progeny of hemizygous *loxP-BphP1* mice using IVIS Spectrum instrument. Top: EGFP fluorescence. Bottom: mCherry fluorescence. **f** Cre recombination in primary hippocampal neurons isolated from homozygous *loxP-BphP1* mouse and transduced with AAV2-Cre, fluorescence images 7 days after transduction. Scale bar, 20 μm. **g** Cre recombination in primary skin fibroblasts isolated from hemizygous *loxP-BphP1* mouse, epifluorescence images 7 days after transduction with AAV6-Cre. Scale bar, 50 μm. **h** IVIS Spectrum in vivo imaging of the progeny of hemizygous *loxP-BphP1* mice and hemizygous Vasa-Cre mice vs homozygous *BphP1-Cre*_*vasa*_ mice. Top: EGFP fluorescence. Bottom: mCherry fluorescence. For more details, see “Statistics and reproducibility” section of the Methods.

The *loxP*-EGFP-*loxP*-BphP1-mCherry-TetR cassette (Fig. 1a) was inserted into *ROSA26* locus, which is generally targeted with high efficiency and supports robust ubiquitous expression. The introduction of a transgene into the *ROSA26* locus did not produce any obvious defects or fertility impairments. While BphP1 expression is sufficient for differential PAT imaging, its fusion with non-mutated (i.e. used in Tet-Off system) TetR tetracycline repressor^27^ should allow transcription activation of genes encoded downstream of TetR-binding nucleotide promoter sequence. mCherry was added to visualize the transgene expression, while EGFP visualized the transgene-containing tissues before Cre-mediated transgene expression.

The offspring were genotyped with two pairs of primers. The first pair of primers amplified the product from the unmodified (wild-type) *ROSA26* locus (Fig. 1b) and did not generate an amplicon when the locus was disrupted by knock-in. The second pair of primers that anneal to the transgene sequence indicated the presence of the transgene in the genome. Thus, both the intact *ROSA26* and transgene sequences are detected in hemizygous animals, only unmodified *ROSA26* locus is detected in wild-type animals, and only transgene sequence is detected in homozygous animals (Fig. 1c, top panel). Crossing of *loxP-BphP1* mice with Vasa-Cre driver mice resulted in excision of EGFP sequence and ubiquitous expression of BphP1-mCherry-TetR protein in progeny (Fig. 1c, middle and bottom panels, and d).

After crossing of hemizygous *loxP-BphP1* mice with wild-type BALB/c mice, F1 progeny inherited the transgene at an expected frequency and expressed EGFP in the skin, as shown by in vivo fluorescence imaging (Fig. 1e). We crossed hemizygous *loxP-BphP1* mice to each other (sibling to sibling) to obtain homozygous mice, which displayed higher EGFP fluorescence (Fig. 1e).

### Cre-dependent expression of BphP1-mCherry-TetR in primary cells

Primary fibroblasts and neurons were further used to test Cre-dependent excision of *loxP*-flanked EGFP coding sequence. Fibroblasts were transduced with adeno-associated virus serotype 6 (AAV6) encoding Cre recombinase under the CAG promoter (AAV6 enables high-efficiency transduction of fibroblasts^28^), while primary hippocampal neurons were transduced with AAV2-CAG-Cre viral particles. Fluorescence imaging performed 7 days after transduction (Fig. 1f, g) demonstrated that a portion of cells has lost EGFP and gained mCherry fluorescence, confirming the Cre-dependent excision of the *EGFP* gene and consequent expression of BphP1-mCherry-TetR fusion. According to flow cytometry analysis, about 65% of skin fibroblasts underwent excision of *loxP*-flanked EGFP (Supplementary Fig. 2).

### Cre-dependent expression of BphP1-mCherry-TetR in vivo

To assess Cre-dependent BphP1-mCherry-TetR expression in vivo and the compatibility of global BphP1 activation with normal survival and postnatal development, hemizygous *loxP-BphP1* males were crossed with females of ubiquitous Cre-driver strain FVB-Tg(Ddx4-Cre)1Dcas/J, also known as Vasa-Cre^29^ (Fig. 1d). In the offspring, as expected by transgene inheritance, a part of animals expressed mCherry (Fig. 1h), confirming that *EGFP* was excised and BphP1-mCherry-TetR is expressed in *ROSA26*^*pCAG-loxP-BphP1-mCherry*^ (hereafter *BphP1-Cre*_*vasa*_) mice. The efficiency of Cre recombination in the offspring can be accessed by the comparison of EGFP fluorescence in the tails of hemizygous *loxP-BphP1* and hemizygous *BphP1-Cre*_*vasa*_ mice (Fig. 1e, h). The hemizygous *BphP1-Cre*_*vasa*_ mice were further crossed to each other to obtain homozygous animals (Fig. 1h).

**Fig. 2:**
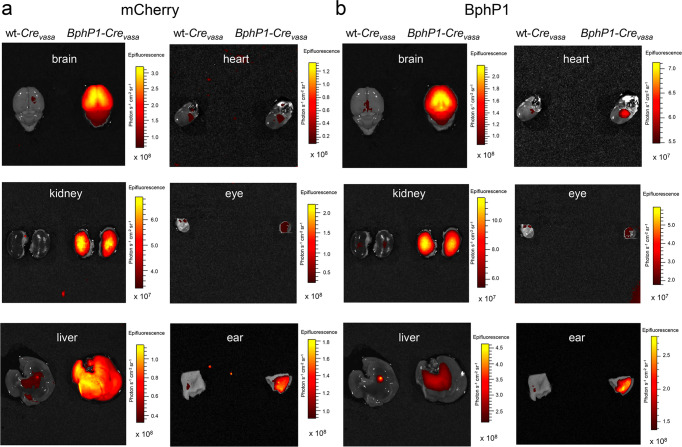
Imaging of wt-*Cre*_*vasa*_ and *BphP1-Cre*_*vasa*_ mouse organs. Mice were euthanized and freshly isolated individual organs were sequentially visualized. Signals were acquired using IVIS Spectrum for (**a**) mCherry fluorescence (Ex. 570/20 nm, Em. 620/30 nm) and (**b**) BphP1 fluorescence (Ex. 675/20 nm, Em. 720/30 nm). Fluorescence images of organs isolated from wt-*Cre*_*vasa*_ (left column) and hemizygous *BphP1-Cre*_*vasa*_ (right columns) mice are shown. For more details, see “Statistics and reproducibility” section of the Methods.

Fluorescence imaging allowed comparing the mCherry signal, as well as naturally weak intrinsic BphP1 fluorescence in the Pr state, in individual organs of hemizygous *BphP1-Cre*_*vasa*_ and wt-*Cre*_*vasa*_ (non-carrier littermates) mice. BphP1 fluorescence was primarily detected in the kidneys, liver, ear (Fig. 2) and brain (Fig. 2 and Supplementary Fig. 3a–c) of *BphP1-Cre*_*vasa*_ mice. Despite the low BphP1 expression in transgenic mice, different levels of its fluorescence in the organs could be due to different concentrations of the endogenous biliverdin chromophore, as well as different rates of BphP1 protein turnover and degradation. Full excision of EGFP in various brain regions and the liver was confirmed by PCR genotyping (Supplementary Fig. 3d). Organs of both *BphP1-Cre*_*vasa*_ and wt-*Cre*_*vasa*_ mice displayed weak green autofluorescence. These data confirm the Cre-dependent expression of the BphP1-mCherry-TetR protein in the *BphP1-Cre*_*vasa*_ mice.

### Optogenetic activation of gene expression in *BphP1-Cre*_vasa_ derived primary cells

**Fig. 3:**
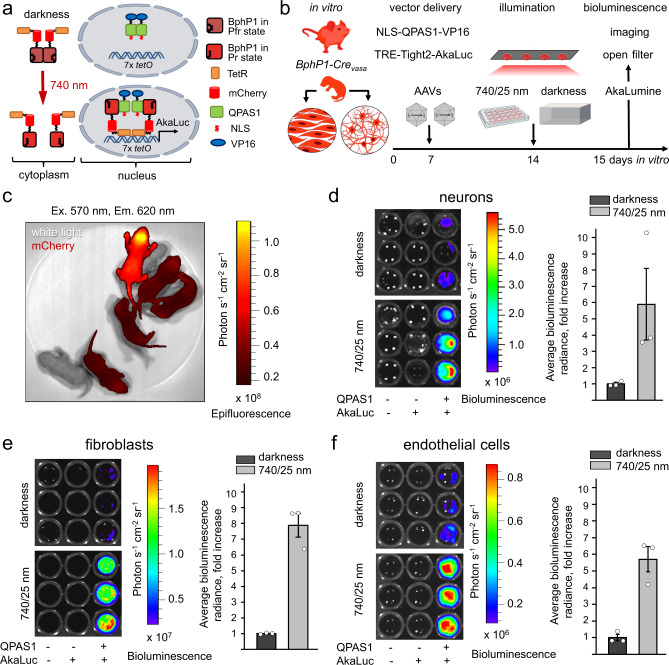
Optogenetic activation of gene transcription in primary *BphP1-Cre*_*vasa*_ derived cells. **a** Schematics of light-induced transcription activation. NIR light converts BphP1 into the Pr state and induces heterodimerization with QPAS1. NLS fused with QPAS1 facilitates translocation of the heterodimer to the nucleus, where BphP1 fusions interact with 7x *tetO* DNA repeats via fused TetR. VP16 fused with QPAS1 recruits the transcription initiation complex and triggers transcription of the AkaLuc reporter gene. **b** Experimental design for optogenetic transcriptional activation in vitro. Primary cultures were isolated from postnatal (P0-P1) homozygous *BphP1-Cre*_*vasa*_ mice. On 7 day in vitro (DIV) cells were transduced with AAVs encoding CAG-NLS-QPAS1-VP16 and TRE-Tight2-AkaLuc (1:5, multiplicity of infection of 10^5^). Seven days after viral transduction cells were illuminated with 740/25 nm light of 0.35 mW cm^−2^ or kept in darkness for 24 h. **c** Selection of homozygous *BphP1-Cre*_*vasa*_ pups based on mCherry fluorescence using IVIS Spectrum imaging. Bioluminescence imaging of the light-induced AkaLuc expression in primary neurons (**d**), fibroblasts (**e**) and endothelial cells (**f**). Before imaging, the culture medium was aspirated, cells were transferred to a black 96-well plate in suspension and supplemented with AkaLumine (250 μM). Visualization was performed in luminescence mode with an open emission filter. Fold increase of AkaLuc-evoked bioluminescence upon illumination with 740/25 nm light was calculated following subtraction of signals produced by a reporter (TRE-Tight2-AkaLuc) only from values obtained with full-component OT. In (**d**–**f**) Data are presented as mean values +/- SEM (*n* = 3; transfection experiments). For more details, see “Statistics and reproducibility” section of the Methods. Source data are provided as a Source Data file.

To study how BphP1-based OTs will work with only two copies of BphP1 gene, providing the low BphP1 expression, we first tested it in primary cultures of cells isolated from different organs of the *BphP1-Cre*_*vasa*_ mice. Primary cortical neurons, skin fibroblasts, and endothelial cells were isolated from *BphP1-Cre*_*vasa*_ postnatal (P0-P1) mice and at 7 days in vitro (DIV) transduced with two AAV PHP.eB delivering QPAS1 binding partner for BphP1 and AkaLuc luciferase reporter gene^30^. QPAS1 was encoded under CAG promoter and fused with VP16 transcription activator (from herpes simplex virus type 1) to attract endogenous polymerase complex, as well as with nuclear localization signal (NLS) to relocate QPAS1 with the bound endogenous BphP1-mCherry-TetR from the cytoplasm to the nucleus (Fig. 3a). *AkaLuc* gene was cloned downstream of a Tet-response element (TRE) in the pAAV-TRE-Tight2 vector. TRE consists of seven copies of *tetO* sequence. The ratio of the AAV PHP.eBs bearing the CAG-NLS-QPAS1-VP16 and TRE-Tight2-AkaLuc was 1:5 (Fig. 2b). As a source of primary cultures, the *BphP1-Cre*_*vasa*_ homozygous pups were distinguished from the hemizygous pups based on the higher mCherry fluorescence using the IVIS Spectrum instrument (Fig. 3c). At 14 DIV, AAV-transduced cells were illuminated for 24 h by a 740/25 nm LED array (0.35 mW cm^−2^) or kept in darkness.

The NIR illumination was expected to cause heterodimerization of BphP1 and QPAS1, subsequent translocation of this light-induced complex to the nucleus and its binding via TetR domain to the *tetO* sequences, resulting in transcription of the downstream *AkaLuc* gene triggered by VP16 transcription activator (Fig. 3a). For detection of AkaLuc expression, adherent cells were detached and resuspended in 100 µl of buffer, followed by the addition of an AkaLumine-HCl, a luciferase substrate that provides NIR emission. The resulting bioluminescence was detected using IVIS Spectrum. The observed light-to-dark ratio of the NIR light-induced BphP1-mediated AkaLuc expression was 5.8-fold for primary neurons (Fig. 3d, 7.9-fold for skin fibroblasts (Fig. 3e, and 5.7-fold for endothelial cells (Fig. 3f). No exogenous biliverdin was added.

### NIR light-induced trigerring of gene expression in vivo

**Fig. 4:**
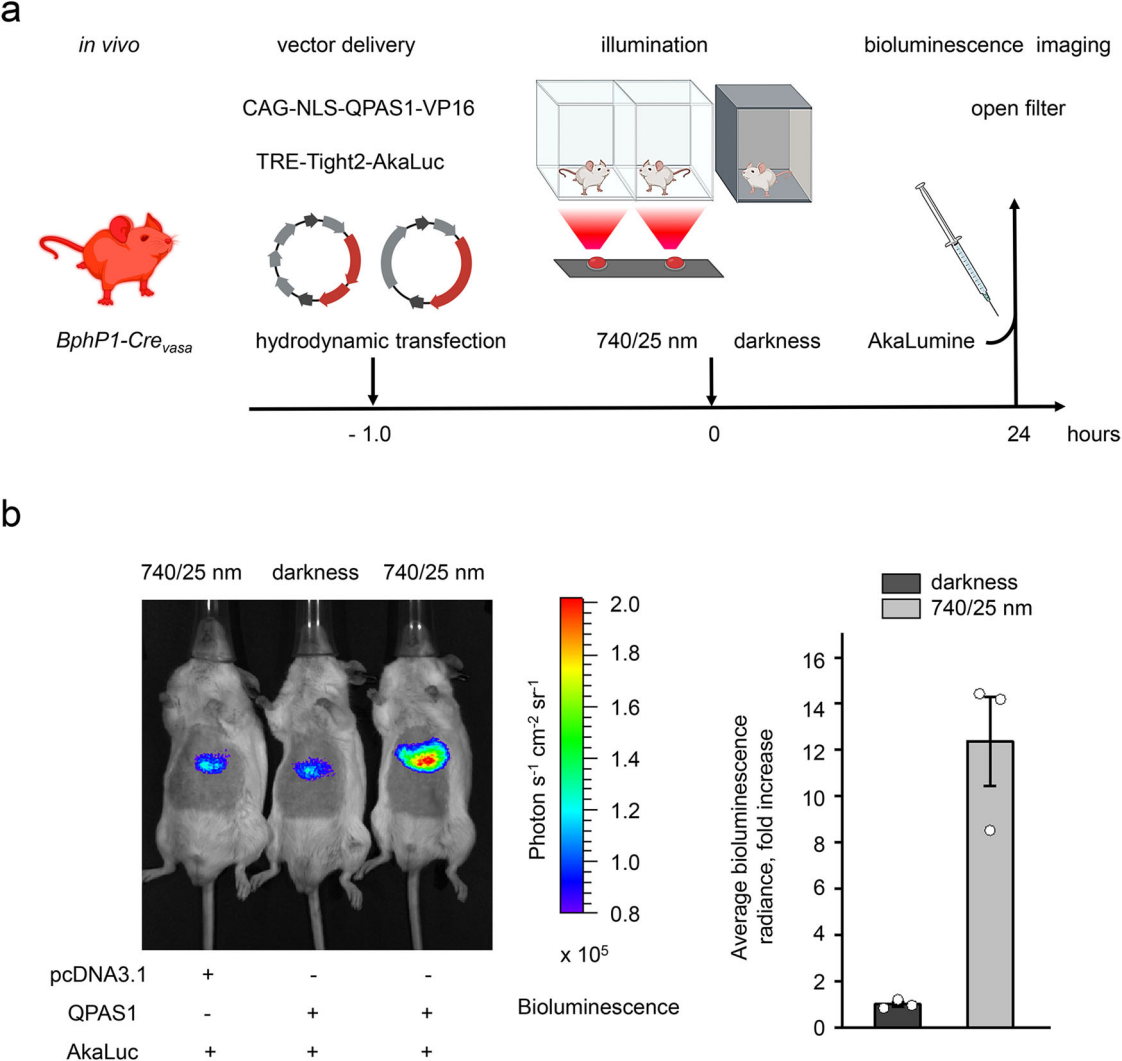
Optogenetic activation of gene transcription in vivo. **a** Experimental design for optogenetic transcriptional activation in *BphP1-Cre*_*vasa*_ mice. Light-induced expression of AkaLuc was detected after hydrodynamic co-transfection with pAAV-CAG-NLS-QPAS1-VP16 (1.7 μg) or empty pcDNA3.1 + (1.7 μg), and pAAV-TRE-Tight2-AkaLuc (8.3 μg) plasmids. Mice were kept in darkness or illuminated with 740/25 nm light of 7.5 mW cm^−2^ for 24 h. Animals were injected with AkaLumine-HCl (75 nmol/g of body weight) 15 min before imaging. **b** In vivo bioluminescence imaging of NIR light-induced expression of AkaLuc in the liver. Visualization was performed in luminescence mode with an open emission filter. The light-to-dark ratio for transcription activation of AkaLuc in the liver was calculated after subtraction of background signal produced by the reporter plasmid only (pAAV-TRE-Tight2-AkaLuc with empty pcDNA3.1 + ). Data are presented as mean values + /- SEM (*n* = 3; independent transfection experiments). Source data are provided as a Source Data file.

We next validated the performance of the BphP1-QPAS1 OT in vivo in the *BphP1-Cre*_*vasa*_ mice. We employed the hydrodynamic tail vein injection for hepatotropic delivery of the OT plasmids, such as pAAV-CAG-NLS-QPAS1-VP16 transactivator and pAAV-TRE-Tight2-AkaLuc luciferase reporter (Fig. 4a). Mice were allowed to recover for 1 h and then were illuminated with a 740/25 nm LED array at 7.5 mW cm^−2^ or kept in darkness for 24 h. Notably, because rodents do not see light above 700 nm^31^, the behavior of the illuminated mice was not disturbed. As a negative control, we illuminated the BphP1-expressing mice injected only with the pAAV-TRE-Tight2-AkaLuc reporter plasmid. Bioluminescence imaging was performed using IVIS Spectrum after injection of AkaLumine-HCl substrate (75 nmol/g of body weight). We found that upon illumination the endogenous BphP1-mCherry-TetR induced 12.4-fold transcription activation of AkaLuc expression in the liver (Fig. 4b). The observed light-to-dark ratio was more than twice higher compared to transcriptional activation mediated by exogenous (plasmid-delivered) BphP1-mCherry-TetR^19^. No exogenous biliverdin was administered to mice.

### Reversibly switchable PAT system for Cre-dependent endogenously-expressed BphP1

**Fig. 5:**
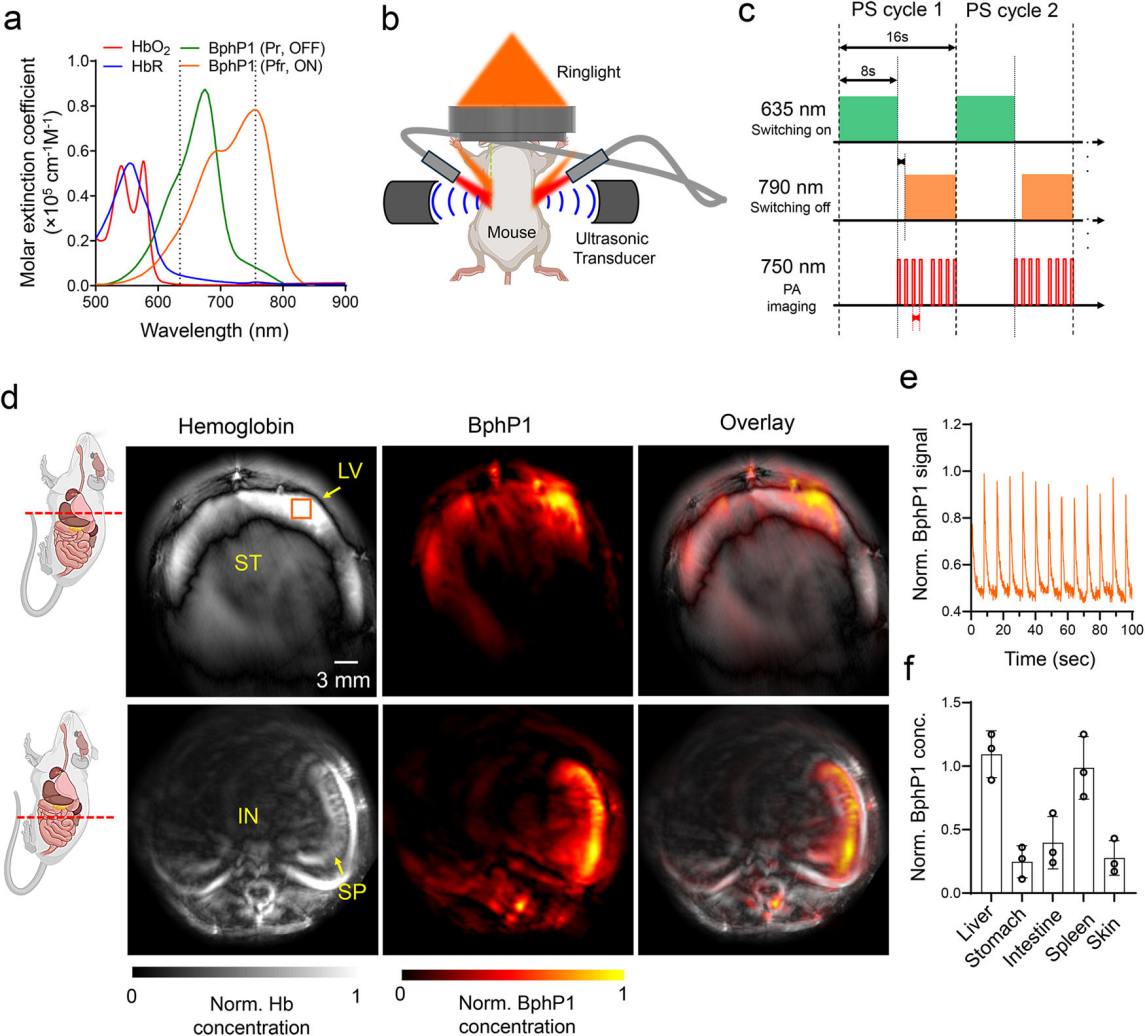
Whole-body PA imaging of *BphP1-Cre*_*vasa*_ mice. **a** Molar extinction coefficient spectra of oxy-hemoglobin (HbO_2_), deoxy-hemoglobin (HbR), ON-state (Pfr) BphP1, and OFF-state (Pr) BphP1. While 635 nm light is used for switching ON BphP1, 750 nm light is used for PS imaging of and switching OFF BphP1. **b** The schematic of the deep PAT system with a ring-shaped ultrasound transducer array. The photoswitching light is delivered by fiber bundles, and the PA imaging light is delivered via free space. **c** The time sequence of the photoswitching and PA imaging. Each cycle contains 8 s of switching-ON continuous-wave light at 630 nm, followed by 8 s of PA imaging at 750 nm. The switching-OFF continuous-wave light at 790 nm is turned on 6 s after the 750 nm light. Please note that the PA imaging light also contributes to switching off BphP1. **d** Differential PAT of the major organs of *BphP1-Cre*_*vasa*_ mouse, showing the different BphP1 intracellular concentrations. IN, intestine; LV, liver; SP, spleen; ST, stomach. **e** The time courses of the BphP1 signals extracted from the liver, showing the reversible photoswitching. Please note that the photoswitching time was not included in the time course of PA imaging. **f** The relative BphP1 intracellular concentration in different organs, normalized by that in the liver. Data are presented as mean values +/- SD (*n* = 3; independent imaging experiments). Source data are provided as a Source Data file.

The key advantage of BphP1 over other PA probes is the reversible photoswitching in the NIR region, which allows differential PA signal detection in deep tissues with higher sensitivity. We upgraded our published reversibly switchable PAT (RS-PAT) system^23^ by increasing imaging speed and detection sensitivity. Two continuous-wave lasers at 635 nm and 790 nm were used for photoswitching BphP1 to the Pfr (ON for PA imaging) and Pr (OFF for PA imaging) states, respectively. A pulsed optical parametric oscillator laser at 750 nm was used for PA imaging of BphP1 transiting from the ON-state to the OFF-state. We chose the PA imaging and photoswitching wavelengths based on the available lasers (pulsed laser for PA imaging and continuous-wave laser for photoswitching) and the absorption spectra of BphP1 at ON and OFF states (Fig. 5a). We chose 750 nm from the optical parametric oscillator laser as the PA excitation wavelength, because it is the peak-absorbing wavelength of the ON-state (Pfr-state) BphP1 (Supplementary Fig. 4a). We chose 635 nm and 790 nm as the photoswitching wavelengths because they are widely available high-power continuous-wave laser wavelengths. Although the photoswitching efficiency at 635 and 790 nm are not optimal as they are not the peak absorbing wavelengths, these industrial-standard continuous-wave lasers can provide strong power, which can compensate for the switching efficiency. We constructed the high-speed PAT system based on a commercial Vesasonics scanner, using a ring-shaped array ultrasound transducer with 512 elements and a ring-shaped light delivery via free space and optical bundles (Fig. 5b, Supplementary Fig. 4b). The tomographic frame rate is 5 Hz, which is 4-fold faster than the previous RS-PAT system. The time sequence of photoswitching and PA imaging of the *BphP1-Cre*_*vasa*_ mice is shown in Fig. 5c, in which each switching/imaging cycle takes 16 s. The differential image was obtained by subtracting the PA image acquired at the beginning (ON-state) and at the end (OFF-state) of each cycle, which effectively eliminated the non-switching background signals from blood. Typically, 24 cycles were acquired and averaged to mitigate the breathing-induced motion artifacts.

### PAT imaging of BphP1 expression

We first validated the system’s performance by imaging a tissue-mimicking phantom made of BphP1-expressing 4T1 tumor cells (Supplementary Fig. 5a). The estimated BphP1 holoform cellular concentration was 2 μM. The PA signal intensity of BphP1 in the ON state was more than 5-fold stronger than in the OFF state. The photoswitching was reversible and repeatable over many cycles without obvious photobleaching (Supplementary Fig. 5b). Given the contrast to noise ratio of the BphP1 signals, the estimated detection sensitivity was ~40 nM.

We then demonstrated the deep imaging capability of PAT using BphP1 as the endogenous contrast agent in *BphP1-Cre*_*vasa*_ mice. The in vivo PA imaging of the whole-body *BphP1-Cre*_*vasa*_ mouse was similar to our previous method, in which the ON-state PA image and the OFF-state PA image are both dominated by the background blood signals, showing the major organs (Supplementary Fig. 6a). The differential image between the ON- and OFF-state shows the BphP1 signals only (Supplementary Fig. 6b). We repeated the imaging experiments on three mice.

We then evaluated the BphP1 expression in different organs. We selected representative cross-sections of the mouse that included the liver, stomach, spleen, and intestine. Differential PA imaging was performed in vivo at each cross-section (Fig. 5d). Robust and repeatable photoswitching was detected in organs, such as the liver (Fig. 5e). The differential PA imaging shows that BphP1 holoform signal in the liver and spleen was higher compared to the stomach (Fig. 5f), reflecting the higher level of endogenous biliverdin in these organs. Comparing the differential signal strength obtained from the *BphP1-Cre*_*vasa*_ mouse and the 4T1 tumor cell phantom, we estimated that the BphP1 intracellular concentration in these organs was ~100 nM. The results have collectively demonstrated the deep penetration and high detection sensitivity of PAT enabled by the BphP1 knock-in mouse model.

### Imaging of developing BphP1-expressing embryo

**Fig. 6:**
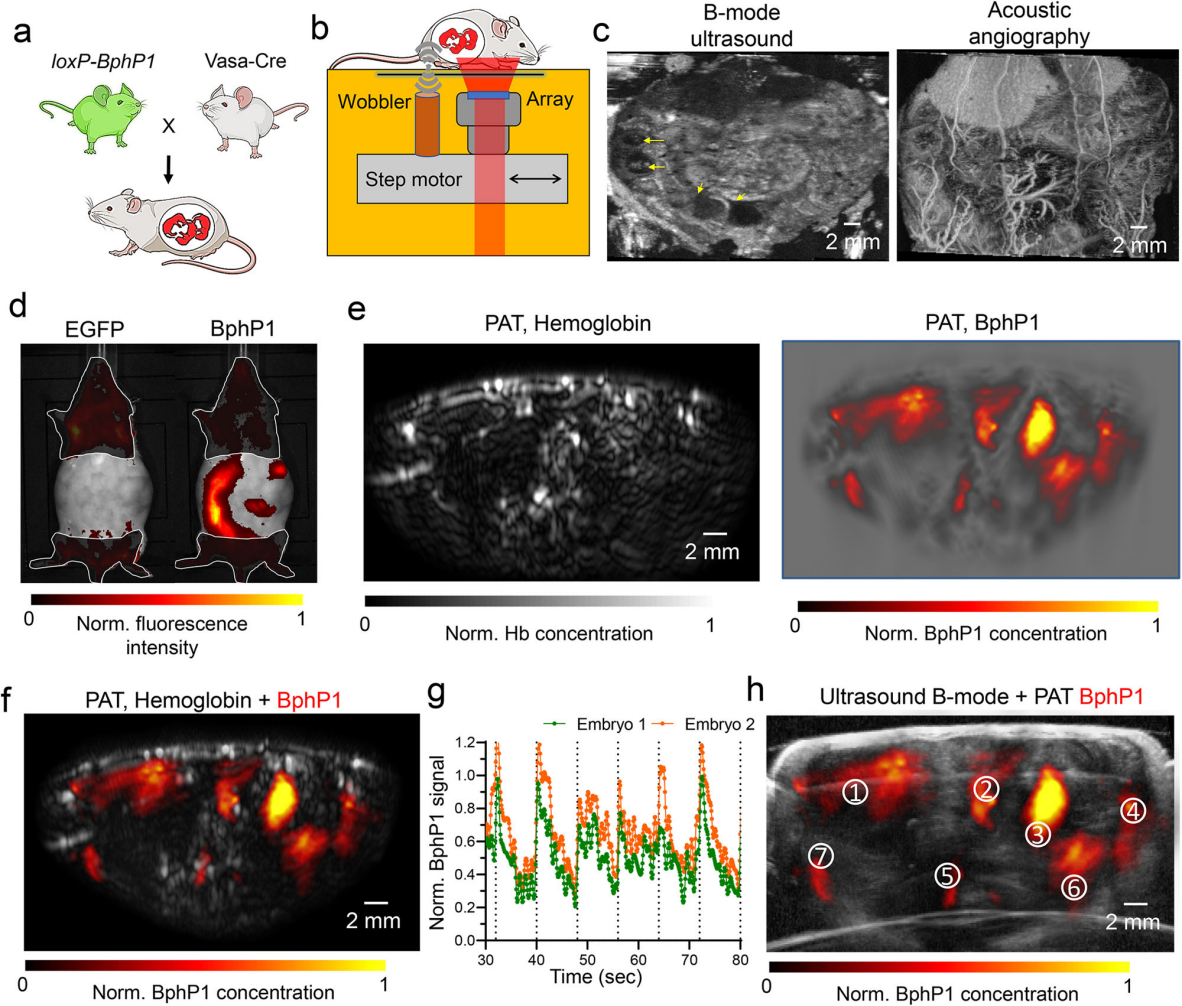
Non-invasive PA imaging of BphP1-expressing embryo in vivo. **a** A *loxP-BphP1* male mouse was crossed with a Vasa-Cre female mouse. All the embryos in the pregnant female mouse were expected to express BphP1. **b** Schematic of the hybrid PA and ultrasound imaging system, in which the linear ultrasound array for PA imaging and the focused wobbler are installed on the same translation motor stage. **c** The ultrasound image and acoustic angiographic image of the pregnant Vasa-Cre mouse show multiple embryos distributed at different depths. **d** IVIS Spectrum images of the pregnant Vasa-Cre mouse, showing no EGFP expression, but BphP1 expression. BphP1 intrinsic fluorescence (Ex. 665 nm, Em. 725 nm) and EGFP fluorescence (Ex. 480 nm, Em. 535 nm). **e** PAT images of hemoglobin signals in the major maternal organs, and BphP1 signals in all seven embryos. **f** Overlayed image of the hemoglobin signals (shown in gray) and the BphP1 signals (shown in color). **g** Robust photoswitching of the BphP1 signals from two embryos. **h** Overlay image of the ultrasound image (shown in grey) and BphP1-expressing embryos (shown in color). For more details, see “Statistics and reproducibility” section of the Methods.

Non-invasive imaging of the developing embryo in vivo is highly desired for developmental and reproductive biology. Traditional high-resolution optical imaging, such as two-photon microscopy, cannot access the deep-seated embryos without implanting intravital windows^32^. We crossed the homozygous *loxP-BphP1* male mouse with the homozygous Vasa-Cre female mouse and imaged the pregnant female mouse on E15 stage (Fig. 6a). To validate the pregnancy and locations of embryos, we developed a hybrid PA-ultrasound system that can provide simultaneous dual-modality imaging of the tissue’s morphological and molecular information (Fig. 6b, Supplementary Figs. 8 and 9). The dual-modality system is also capable of acoustic angiography of the blood vessels by using gas-filled microbubbles as the contrast agent (Supplementary Fig. 9). The ultrasound imaging utilizes a high-frequency focused ultrasound transducer and can provide the embryo morphology and location (Fig. 6c). From the ultrasound image, we confirmed there were a total of seven embryos in the field of view with various sizes at depths ranging 1–7 mm beneath the abdominal skin surface. The BphP1 expression in the embryos was confirmed by the IVIS Spectrum results (Fig. 6d).

We then performed the differential PA imaging of the pregnant dam at approximately the same location as the ultrasound imaging. As expected, the traditional PA images were dominated by the blood signals throughout the mouse (Fig. 6e), and it was challenging to differentiate the embryos from the maternal organs. By contrast, the BphP1 signals obtained by the differential PA imaging were detected only from the embryos, but not from the maternal organs (Fig. 6f). Robust and repeatable photoswitching were observed on the embryos, with a switching ratio ranging from 2.5 to 5. All the embryos identified by the ultrasound imaging were expressing BphP1 (Fig. 6h), with consistent locations between two imaging modalities. We also observed that the BphP1 intracellular concentrations varied substantially from individual embryos, likely because of their different orientations.

### Tracking viral-mediated Cre-dependent BphP1 expression

**Fig. 7:**
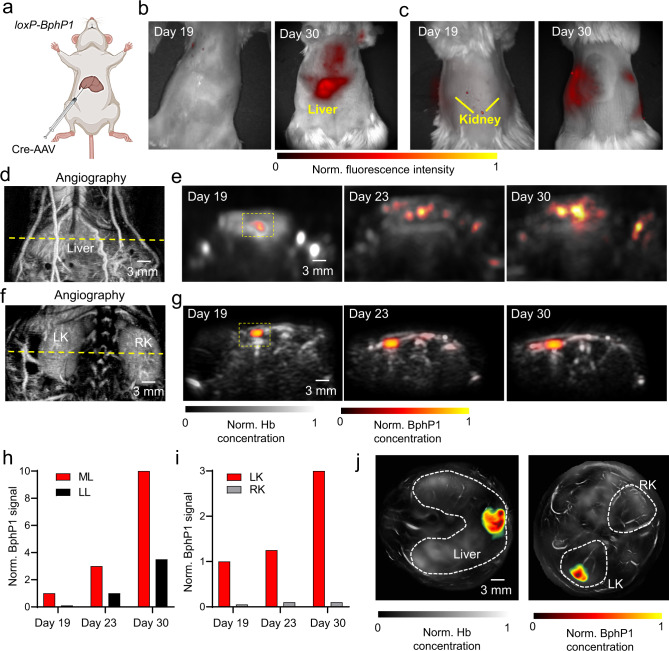
Longitudinal PA imaging of the viral-mediated Cre-dependent specific expression of BphP1 in vivo. **a** Cre-expressing AAVs was injected into the median liver lobe (AAV8) or a left kidney (AAV2) of the *loxP-BphP1* mouse. **b**, **c** IVIS images of the mouse after the AAV injection into the liver (**b**) and left kidney (**c**), showing the BphP1 expression on Day 30. **d** Acoustic angiography image of the mouse liver region, acquired by the dual-modality ultrasound and PA imaging system. The dashed line marks the approximate region monitored by longitudinal PA imaging. **e** PA images of the BphP1 signals (shown in color) in the mouse liver at days 19, 23 and 30 after the AAV injection, acquired by the dual-modality system. The background hemoglobin signals were shown in grey. **f** Acoustic angiography image of the mouse kidney region. The dashed line marks the region monitored by PA imaging. **g** PA images of the BphP1 signals (shown in color) in the mouse left kidney at days 19, 23, and 30 after the AAV injection. There was no BphP1 expression observed in the right kidney. **h** Quantitative analysis of the mean BphP1 signals in the liver at different time points, as indicated by the dashed boxes in (**e**). ML, median lobe; LL, left lobe. The mean is the averaged pixel value in the dashed box. **i** Quantitative analysis of the mean BphP1 signals in the kidneys at different time points, as indicated by the dashed boxes in (**e**). LK, left kidney; RK, right kidney. The mean is the averaged pixel value in the dashed box. For **h**, **i** *n* = 2 biological replicates). **j** PA images of the BphP1 signals (shown in color) around the liver and kidney region of the same mice, acquired by the ring-array-based PA imaging system. The background hemoglobin signals are shown in grey. Source data are provided as a Source Data file.

We next evaluated the longitudinal PAT of viral-mediated Cre-dependent BphP1 expression in the *loxP-BphP1* mouse at larger depths. We injected the Cre-expressing AAV8 into the mouse’s median liver lobe or Cre-expressing AAV2 into the left kidney (Fig. 7a) and used the left liver lobe or right kidney as the controls. PA imaging was performed starting from day 19 after the AAV injection, using the dual-modality PA-ultrasound imaging system. The BphP1 expression in the liver and kidney was confirmed by fluorescence imaging on day 30 (Fig. 7b, c). We first used microbubble-enhanced acoustic angiography to identify the liver (Fig. 7d) and the kidney (Fig. 7f), and then used BphP1 PA imaging to monitor approximately the same liver and kidney regions at days 19, 23, and 30 (Fig. 7d, f).

According to the spread of the virus in the liver, the BphP1 expression was first detected at the median lobe by day 19, and the intracellular concentration was elevated by 3-fold on day 23 and 10-fold on day 30 (Fig. 7e, h). By day 30, the BphP1 expression was also observed in the left liver lobe, indicating further spreading of AAV. For the kidney, the BphP1 expression was confined in the left kidney throughout the experiment. Compared with day 19, the BphP1 expression was elevated by 2-fold on day 23 and 3-fold on day 30 (Fig. 7g, i). There was no detectable BphP1 expression in the right kidney.

**Fig. 8:**
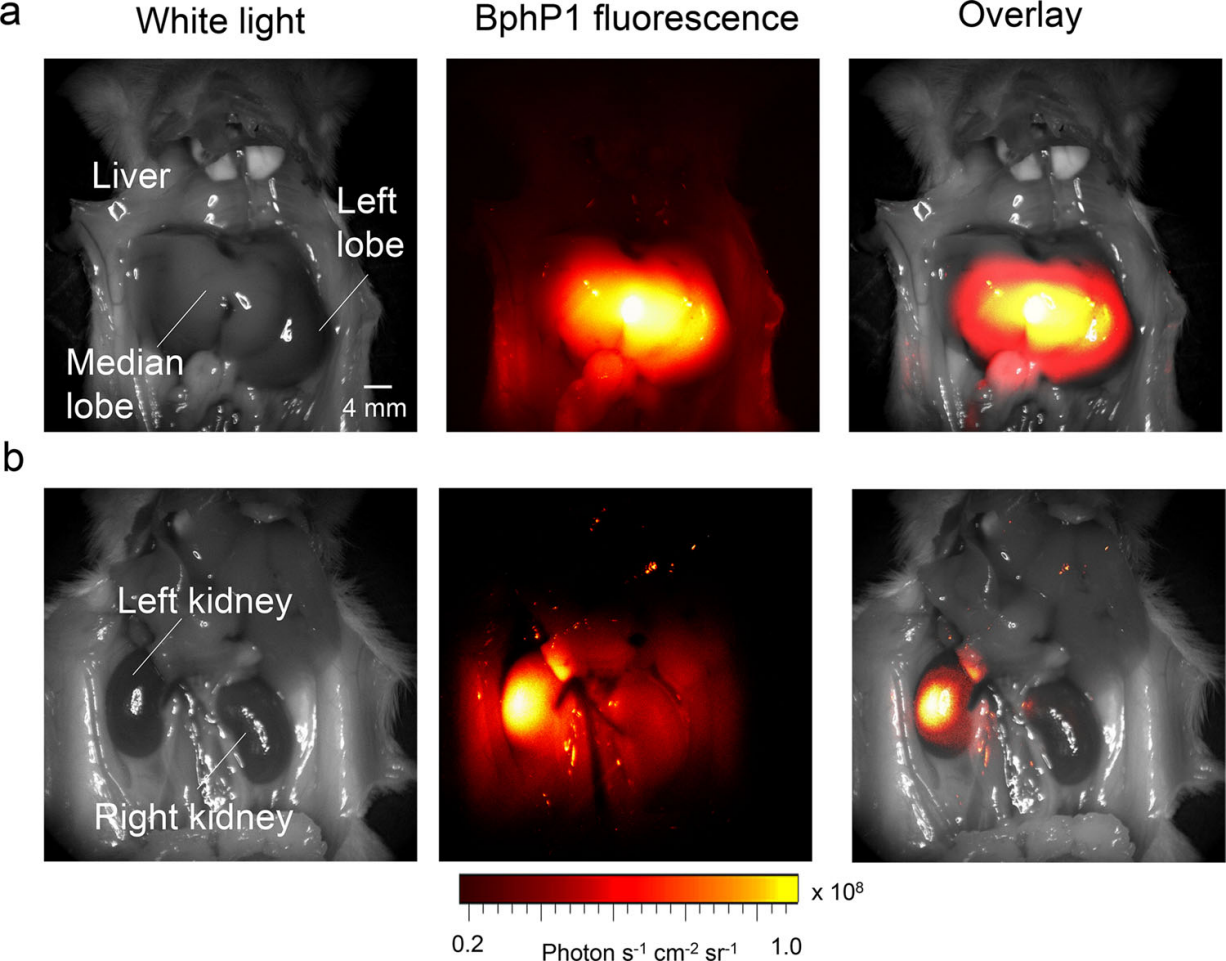
Fluorescence imaging of the AAV-mediated Cre-dependent BphP1 expression. **a** White-light and BphP1 fluorescence images (Ex. 665 nm, Em. 725 nm) of the liver region show the high BphP1 intracellular concentration in the median liver lobe. **b** White-light and BphP1 fluorescence images (Ex. 665 nm, Em. 725 nm) of the kidney region show the high BphP1 expression level in the left kidney. Images in (**a**), (**b**) were acquired using IVIS Spectrum on day 30 after the AAV-Cre transduction.

Notably on day 19, in contrast to PA imaging, our fluorescence imaging was not able to detect signals in the deep tissue of mice, like liver or kidney. We further imaged the BphP1 expression in the liver and left kidney at day 30, using the ring-array-based PA system, which can provide improved imaging quality but lower imaging speed (Fig. 7j). The ring-array PA results have confirmed that the liver had stronger BphP1 expression than the kidney. The end-point fluorescence imaging with the exposed organs clearly shows the BphP1 expression in the median liver lobe and the left kidney (Fig. 8). Overall, these results demonstrated the longitudinal PA imaging of the Cre-dependent BphP1 expression in the *loxP-BphP1* mouse model, with large imaging depth and high detection sensitivity.

### Monitoring of liver regeneration after partial hepatectomy

**Fig. 9:**
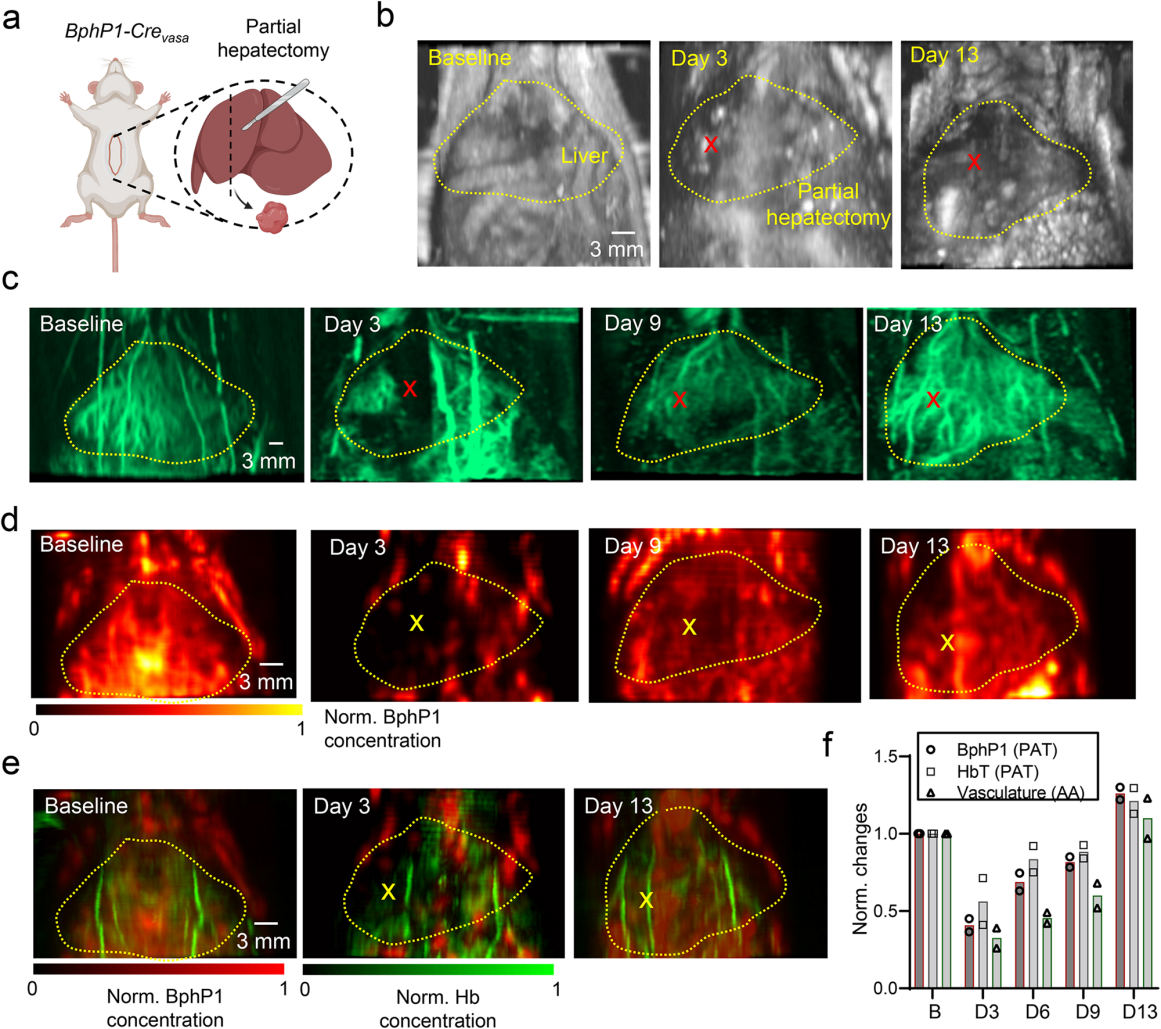
Longitudinal PA imaging of the liver regeneration after partial hepatectomy. **a** Partial hepatectomy was performed on the median liver lobe of the BphP1-expressing mouse. Approximately 50–75% of the lobe was cut. **b** The ultrasound images of the mouse liver region before the surgery, and at days 3 and 13 after the surgery. The yellow dashed lines outline the liver region, and the red crosses mark the approximate location of liver cutting. **c** Acoustic angiography images of the blood vessels in the same liver regions before, and at days 3, 9, and 13 after the surgery, showing the liver tissue regeneration. The crosses mark the locations of resection and regenerated tissues. **d** PA images of the BphP1 signals expressed by the liver cells before and after the surgery, showing the liver tissue regeneration. **e** Overlapped BphP1 signals (shown in red) and the hemoglobin signals (shown in green) in the mouse liver before and after the surgery. **f** Quantitative analysis of BphP1 signals and hemoglobin signals measured by PAT, as well as the vasculature volume measured by acoustic angiography before and after the surgery. The region of interest for quantitative analysis is indicated by the dashed boxes in (**c**–**e**). Error bars, s.d. (*n* = 2; independent imaging experiments). Source data are provided as a Source Data file.

We next demonstrated a longitudinal PAT study of the liver regeneration process, using BphP1 expressed by the proliferating liver cells as the endogenous contrast. Mouse liver regeneration after partial hepatectomy is a complex process, in which the remaining liver recovers to its initial size in approximately 10 days. Understanding the mechanisms of liver regeneration is useful for liver resections or transplantation, and may also be beneficial for transplantology, surgical oncology and regenerative medicine. In this experiment, we used the dual-modality PA-ultrasound imaging system to monitor liver regeneration in vivo based on genomically expressed BphP1. We acquired the baseline images of the mouse liver, and then surgically removed ~50–75% of the median lobe of the liver (Fig. 9a). The mice were then longitudinally monitored up to 13 days after the surgery. We repeated the imaging experiments on two mice.

While the B-mode ultrasound images have relatively low contrast of the liver region (Fig. 9b), the acoustic angiography images captured the loss of vascularization in the resection region after the hepatectomy and its gradual restoration following regeneration (Fig. 9c). The angiography results have confirmed the substantial reduction of blood vessels 3 days after the surgery, and the complete recovery to the baseline level after 13 days. Similar to the imaging of viral-mediated BphP1 expression, we applied photoswitching PAT to monitor the BphP1-expressing liver tissue regeneration (mostly hepatocytes) (Fig. 9d), which followed approximately the same timeline as the angiography results. In the PAT images, the BphP1 signals in the median lobe of the liver were much weaker 3 days after the surgery, when the liver regeneration process was still in the priming phase. The BphP1 signals recovered to the baseline level 13 days after the surgery, indicating the full replacement of the lost liver tissue. We also separated the BphP1 signals of the liver cells and the hemoglobin signals of blood vessels and then overlapped them to demonstrate their distinguished patterns in the liver region (Fig. 9e). We performed quantitative analysis of the BphP1 and hemoglobin signals measured by PAT, as well as the vasculature volume measured by acoustic angiography (Fig. 9f). All three measurements have shown a consistent tissue regeneration timeline and could be used for assessing complementary aspects of the liver regeneration process, such as angiogenesis and proliferation.

## Discussion

We have developed a transgenic mouse model with a knocked-in BphP1 soluble bacterial NIR photoreceptor. In contrast to available mouse strains with conditional expression of various fluorescent proteins including iRFPs, the developed in this study *loxP-BphP1* mouse enables both, spatiotemporal optogenetic regulation and PA imaging in deep tissues. These different types of applications are possible using the same genomically integrated BphP1 construct.

BphP1-mCherry-TetR can be expressed in distinct tissues or cell subtypes by using viral-vector-mediated Cre-recombination or breeding with Cre-driver mice, in which the Cre recombinase production is restricted to a certain tissue or even is chemically inducible^33,34^. This enables conditional transgene targeting and provides the cell or tissue specificity for optogenetic manipulation and PA imaging. Yet additional spatiotemporal optogenetic regulation can be achieved with focused light that further confines the manipulation area. Among several Cre-driver mouse strains for ubiquitous recombination of the BphP1 construct, including CMV-Cre^35^ and Ella-Cre^36^, we chose the Vasa-Cre mouse females, which provide global irreversible recombination in progeny due to perdurance of Cre recombinase in zygote^29^.

The endogenously produced BphP1 preserves its functionality in various mouse cell types and tissues, enabling multiple photoswitching cycles between its Pfr and Pr absorbing states. However, the concentration of BphP1 assembled with biliverdin chromophore (holoform) varied among different organs and tissues. There are several possible reasons for that including different levels of endogenous biliverdin; possible higher stability of BphP1 holoform than apoform, which has been observed for BphP-derived NIR FPs^37^, resulting in holoform accumulation in cells with the higher biliverdin concentration; naturally distinct among tissues turnover rate of all proteins, affecting BphP1 too; and distinct division rates of cells of different origins. The latter can also contribute to uneven BphP1 signals among cells in the same organ.

The ground Pfr state of BphP1 was used as an ON-state for the differential PA imaging, whereas the photoswitched Pr state enabled binding of the QPAS1 protein partner for the optogenetic transcription regulation. We demonstrated that the BphP1-QPAS1 OT enables efficient optogenetic gene transcription activation in the *BphP1-Cre*_*vasa*_ mice and derived primary cells of both mesodermal and ectodermal origins. Because the QPAS1 component is delivered exogenously, it can be expressed under various promoters to further improve spatial targeting of optogenetic manipulation. In addition, the use of specific shRNAs as a reporter in this OT will repress gene transcription at the desired stage of development or progression of the disease and for a certain time. Thus, the *loxP-BphP1* mouse can be applied to both, reversible activation and repression of gene transcription.

We developed two advanced PAT systems for the whole-body differential BphP1 imaging and observed the BphP1 expression in major organs, developing embryos and regenerating liver of the *BphP1-Cre*_*vasa*_ mice, as well as in AAV-Cre infected liver and kidney, with a maximum penetration depth exceeding 1 cm and detection sensitivity of tens of nanomoles. Our imaging modality provides deep tissue visualization with clearly distinguishable BphP1 and hemoglobin signals, as well as volumetric studies of vasculature in living mice, which allows versatile monitoring of tissue development and renewal.

The wavelength-dependent light attenuation leads to different optical fluence and thus penetration depth in tissues, which is the reason for low quantitative accuracy and low detection sensitivity of the traditional spectral unmixing method in PA imaging of the genetically encoded probes. By contrast, our BphP1 photoswitching-based PA technique is not sensitive to the wavelength-dependent light attenuation and penetration depth, because the differential imaging is performed on the ON- and OFF-state images acquired at the same wavelength (e.g., 750 nm in our experiments). The only requirement in the photoswitching process is that the switching light is strong enough to fully switch the BphP1 to the ON state, which is readily achievable by using the strong continuous-wave laser used in our experiments.

In this study, we used the in vitro measurements to estimate the in vivo detection sensitivity of BphP1 intracellular concentration, which is not ideal but the most practical approach we have demonstrated previously^23^. One alternative way to validate the in vivo concentration of the BphP1 is to isolate the cells, purify the protein, and quantify the averaged concentration. Nevertheless, this approach is not capable of mapping the spatial inhomogeneity of different cell types in the same organ.

With the constantly increasing number of Cre-driver mouse strains^34^ and the development of AAVs of various tissue tropism^38^ encoding Cre under tissue-specific promoters, the *loxP-BphP1* knock-in mouse will become an essential research model for precise PA imaging and optogenetically-controlled gene expression. We envision its future use in a wide range of studies including deep-tissue imaging and optogenetic manipulation of tissue development and morphogenesis, the fate of cell populations, dynamics of allograft transplants, tissue regeneration, tumor progression, and the spread of infection, among many others.

## Methods

### Ethical statement

All procedures with animals were performed in an AAALAC-approved facility and received ethical approval from the Institutional Animal Care and Use Committee of the Albert Einstein College of Medicine (protocol 00001050) and Duke University (protocol A009-20-01).

### Housing and husbandry

Mice were maintained on a 12 h light and dark cycle at room temperature and standard humidity (~40–55%), with ad libitum access to food and water. Same-sex littermates were housed together in cages with chopped corn cob bedding (2–5 mice per cage). Environmental enrichment included pieces of compressed cotton nestlets and paper huts.

### Molecular cloning

A vector backbone pROSA26-CAG was used for targeting vector construction. Cassette encoding transgene was assembled using as a backbone pCALNL-EGFP (Addgene#13770). The first vector was digested with EcoRI and NotI and ligated with linker DNA having *NheI*, *HindIII* and *BglII* sites to delete EGFP and create a multicloning site. Next nucleotide sequence encoding Neomycin was substituted by sequence with *KpnI* and *EcoRI* sites using the QuikChange kit. Then EGFP was inserted between two *loxP* sequences by *KpnI* and *EcoRI* restriction sites. The resulting vector was digested by *NheI* and *NotI* and ligated with *NheI*-*BglII* fragment of pKA-207I10 (Addgene #79845) encoding BphP1-mCherry-TetR and PCR amplified from Ai65 (Addgene #61577) sequence of WPRE-bGHpolyA digested with *BglII* and *NotI*. The final assembly of the targeting vector where the transgene was cloned between homologs arms for knock-in into the *ROSA26* locus was performed by Ingenious Targeting Laboratory.

The targeting vector can be linearized using *NotI* enzyme before electroporation. The total size of the targeting construct (including the backbone vector) is about 19.2 kb. The pCAG-*loxP*-EGFP-pA-*loxP*-BphpP1-mCherry-TetR-WPRE vector was designed such that the expression of EGFP or BphP1-mCherry-TetR-WPRE cassette is driven by the CAG promoter in the *ROSA26* locus (Fig. 1a). The EGFP reporter gene is flanked by *loxP* sites, which enable irreversible Cre-induced excision of EGFP and expression of BphP1-mCherry-TetR-WPRE cassette. FRT-flanked Neo selection cassette is followed immediately downstream of the knock-in cassette. The targeting vector contains a short homology arm (SA) with 1.09 kb *ROSA26* genomic sequence upstream of the pCAG promoter and a 4.34 kb long homology arm (LA) downstream of the Neomycin cassette. The targeting vector was confirmed by restriction enzyme analysis and sequencing after each modification step. The boundaries of the pCAG and the Neo cassette were confirmed by sequencing with primers ROSASQ1 and ROSASQ2.

To generate a pAAV-TRE-Tight2-AkaLuc plasmid, the fragment encoding AkaLuc^30^ was amplified from pBAD-AkaLuc plasmid^39^ with 5′-GTCGGATCCGCCACCATGGAAGATGCCAAAAACATTAAG-3′ and 5′-GTCGCGGCCGCTTACACGGCGATCTTGCCGTCC-3′ primers and subcloned into pTRE-Tight2 plasmid (Addgene #19407) using *BamHI* and *NotI* restriction sites. The fragment encoding TRE-Tight2-AkaLuc was then amplified with 5′-GTGGGCTAGCTAATGTGAGTTAGCTCACTC-3′ and 5′-TTATCTCGAGTTACACGGCGATCTTGCCG-3′ primers, and fragment encoding AAV backbone was amplified from pAAV-CW3SL-EGFP (Addgene #61463) plasmid using 5′-TCCGCTCGAGATAATCAACCTCTG-3′ and 5′-ATCGCTAGCCCACAAGTGTGCAGCATCT-3′ primers. The fragments were combined using *NheI* and *XhoI* restriction sites.

To generate a pAAV-CAG-NLS-QPAS1-VP16 plasmid, two deletions were introduced into the pCMV-NLS-PpsR2-VP16^19^ plasmid using QuikChange mutagenesis kit (Agilent Technologies) with 5′-CAAAAGGAGGAAGACGTCTCCACCGGGCAAGAACATGCAGGCGGTC-3′, 5′- GACCGCCTGCATGTTCTTGCCCGGTGGAGACGTCTTCCTCCTTTTG-3′ and 5′-CGACTCCCGCGATCGACGACGACGACAAAGGTGTCGTTGCTTCTGC-3′, 5′-GCAGAAGCAACGACACCTTTGTCGTCGTCGTCGATCGCGGGAGTCG-3′ pairs of primers. The fragment encoding NLS-QPAS1-VP16 was then amplified using 5′- GTCGGTACCGCCATGTCGCGGAGGCGGCAC-3′ and 5′- GTCGAATTCTTACCCACCGTACTCGTCAATTCC-3′ primers and subcloned into a pAAV-CAG-mRuby2 plasmid (Addgene #99123) using *KpnI* and *EcoRI* restriction sites.

### Generation of transgenic mice

Hemizygous BALB/c mice, carrying pCAG-*loxP*-EGFP-*loxP*-BphP1-mCherry-TetR transgene in *ROSA26* locus (*loxP-BphP1* mice) were generated by Ingenious Targeting Laboratory. Specifically, 10 μg of the targeting vector were linearized and then delivered by electroporation into BALB/c embryonic stem cells (iTLb). After selection with G418 antibiotic (Neomycin analog), surviving clones were expanded for PCR analysis to identify recombinant ES clones. Sequencing was performed on purified DNA to confirm the 5′ genome/pCAG junction. Transgene-carrying cells were inserted into murine blastocysts.

The founder hemizygous *loxP-BphP1* mice were crossed with wild-type BALB/c mice (BALB/cAnNHsd, Envigo). The presence of the transgene in the offspring was detected using PCR. Ear punches (~1 mm in diameter) were incubated with 250 μl of 10 mM NaOH, 0.1 mM EDTA at 95 °C for 10 min. One microliter or solution was used as a template for PCR. The thermal protocol consisted of initial hold 95 °C – 1 min, followed by 35 cycles of 95 °C for 15 s, 58 °C for 20 s, 72 °C for 30 s, and additional 72 °C for 2 min. The reaction volume was 20 μl. PCR products were visualized using agarose gel electrophoresis. The insertion of the transgene into *ROSA26* was detected using PCR with primers 5′-CCAGAGGCCACTTGTGTAGC-3′ and 5′-TGCTTACATAGTCTAACTCGCGAC-3′, annealing to the junction between transgene and the long arm of *ROSA26*. In the presence of transgene, a 494 bp DNA fragment was amplified.

The second-generation hemizygous animals were crossed (sibling to sibling) to obtain homozygous offspring. Their genotypes were identified using PCR with primers targeted to insert *EGFP* sequence (5′-GGCAGAGGATCGTTTCGCGG, 5′-GAAGCACTGCACGCCGTAGG, amplicon size 240 bp) and primers targeted to wild-type *ROSA26* locus (5′-AGCACTTGCTCTCCCAAAGTC, 5′-TGCTTACATAGTCTAACTCGCGAC, amplicon size 564 bp). Uncropped and unprocessed scans of agarose gels with PCR amplification products are provided in the Source Data file. Only 240 bp product was amplified in samples from homozygous animals. Homozygous animals were crossed to each other (sibling to sibling) to maintain a colony.

To confirm the Cre-mediated recombination the F1 progeny of *loxP-BphP1* males and Vasa-Cre (strain FVB-Tg(Ddx4-Cre)1Dcas/J, Jackson Laboratory, JAX #006954) females were genotyped using primers targeted to the *EGFP* sequence and primers targeted to the *BphP1* sequence (5′-GAGCGCGTCCACACCGTTAC, 5′-GGCAACATGTCGCGGAACGA, amplicon size 341 bp). Following breeding of hemizygous *BphP1-Cre*_*vasa*_ mice, homozygous animals were generated and their genotypes were discriminated using primers targeted to the transgene *BphP1* sequence and wild-type *ROSA26* locus.

### Preparation of high-titer AAVs

AAV PHP.eB particles encoding Tight2-AkaLuc under TRE promoter and NLS-QPAS1-VP16 under CAG promoter were prepared as described^40^. Briefly, a plasmid for AAV production was purified with NucleoBond Xtra Maxi EF kit (Macherey-Nagel) and AAV-293T cells (Agilent) were co-transfected with the pAAV-CAG-NLS-QPAS1-VP16 or pAAV-TRE-Tight2-AkaLuc plasmids, a pPHP.eB Rep-Cap plasmid and a pHelper plasmid (TakaraBio) using polyethyleneimine (Santa Cruz). Cell growth medium was collected 72 h after transfection. 120 h after transfection cells and growth medium were collected and combined with medium collected at 72 h. Cells were pelleted by centrifugation and then lysed with salt-active nuclease (SAN, ArcticZymes). 8% PEG was added to the supernatant, incubated for 2 h on ice and then pelleted by centrifugation. PEG pellet was treated with SAN, combined with lysed cells and clarified by centrifugation. The supernatant was applied on an iodixanol gradient and subjected to ultracentrifugation for 2 h 25 min at 350,000 g. Virus fraction was collected, washed and concentrated on Amicon-15 100,000 MWCO centrifuge devices (Millipore). Purified virus aliquots were stored at −80 °C. Virus titer was defined by qPCR. For this, virus aliquots were consequently treated with micrococcal nuclease (New England Biolabs) and proteinase K (Fermentas) and then used as a template for qPCR. NheI digested AAV plasmids with known concentrations were used as standards for titer calculations.

AAV2, AAV6 and AAV8 encoding Cre recombinase was purchased from the AAV Gene Transfer and Cell Therapy Cora Facility of the University of Helsinki (Finland).

### Isolation of primary cultures and AAV transduction

Primary cultures of neurons, fibroblasts and endothelial cells were isolated from homozyhous *BphP1-Cre*_*vasa*_ mice (both males and females). Neurons were isolated from hippocampi of postnatal (P0-P1) mice using the published protocol^41^ and cultured in Neurobasal Plus Medium with B-27 Plus Supplement (Gibco), additional 1 mM GlutaMAX (Gibco, 35050061), 100 U/ml penicillin and 100 μg/ml streptomycin, on poly-D-lysine (EMD Millipore) coated glass coverslips (thickness 0.13–0.17 mm, diameter 12 mm, ThermoFisher Scientific) at density ~70,000 cells per coverslip. Half of the medium was exchanged twice a week.

Primary fibroblasts were isolated from the skin using protocol from^42^, with the following modifications. Digestion was performed in DMEM with 10% FBS, 100 U/ml penicillin and 100 μg/ml streptomycin, 0.25% collagenase, 15 mM HEPES pH7.4 at 37 °C for 1.5 h, while shaking at 220 rpm. Cells were cultured in DMEM with 10% FBS, 100 U/ml penicillin and 100 μg/ml streptomycin. Upon reaching confluency the fibroblasts were dissociated with a trypsin-EDTA solution and replated at a 1:5 ratio. Under these conditions, the fibroblasts might be cultured for up to 6 passages (~20 days).

Endothelial cells were isolated from mouse aortas as previously described^43^. Aortas were dissociated with trypsin/EDTA solution through a 25 G needle. Cells were grown in DMEM containing 2% FBS, 100 U/ml penicillin, 100 μg/ml streptomycin, 0.1 mmol/L citrate at 37° and used for up to 5 passages. Cells were allowed to reach 70% confluency before transduction.

Depending on the experiment, primary cells were transduced at 7 days in vitro (DIV) with AAV2-Cre or AAV PHP.eBs at the multiplicity of infection of 10^5^.

### Fluorescence microscopy

Primary neurons were imaged with Olympus IX81 inverted epifluorescence microscope, operated with a SlideBook v.6.0.8 software (Intelligent Imaging Innovations). Primary fibroblasts were imaged using Olympus IX81 microscope operated with μManager software^44^. Images were taken using Orca Flash 4.0 LT camera (Hamamatsu). The following LED light sources were used for fluorescence excitation (all from Mightex Systems): 480 nm for EGFP and 570 nm for mCherry.

### Flow cytometry

Primary skin fibroblasts grown in a 24-well plate were detached with trypsin (10 min at 37 °C) and resuspended in an ice-cold cell sorting buffer (PBS with 2% FBS and 5 mM EDTA). The cell suspension was analyzed on the BD LSRII flow cytometer. Data were collected using BD FACSDiva v.8.0.1 (BD Biosciences) software. For EGFP fluorescence measurement 488 nm laser, 525/50 excitation filter, 505DLP dichroic were used. For mCherry fluorescence measurement 561 nm laser, 610/20 excitation filter, 600DLP dichroic were used.

### Hydrodynamic transfection of the liver

Homozyhous *BphP1-Cre*_*vasa*_ mice (6–10 months of age) were used in the experiments (total 9 mice, *n* = 3 for each of 3 tested groups). Both males and females were included in the analysis (6 males, 3 females) with only same-sex animals taken within experiment. For hydrodynamic transfection of the liver, 1.7 μg of pAAV-CAG-NLS-QPAS1-VP16 (or empty pcDNA3.1+ plasmid) with 8.3 μg of pAAV-TRE-Tight2-AkaLuc reporter plasmid in 1 mL PBS were intravenously injected through the tail vein within 5 s. After injection, all manipulations with mice kept in darkness were performed under a 530 nm safelight.

### Optogenetic activation with NIR light

On 7 day in vitro (DIV) primary cells were transduced with AAVs encoding CAG-NLS-QPAS1-VP16 and TRE-Tight2-AkaLuc (1:5, multiplicity of infection of 10^5^). Seven days after viral transduction cells were illuminated with 740/25 nm light of 0.35 mW cm^−2^ or kept in darkness for 24 h (Fig. 3b). For in vivo experiments, *BphP1-Cre*_*vasa*_ mice were placed in custom-made transparent cages and illuminated from the bottom with a 740/25 nm LED array with the intensity of the activation light 7.5 mW cm^−2^ (Fig. 4a). For better illumination and imaging, the belly fur was shaved with trimmer. Control animals were kept in conventional cages in complete darkness. Mice were released every 12 h in conventional cages with free access to food and water for 45 min.

### In vivo and ex vivo fluorescence imaging

Fluorescence and bioluminescence imaging was performed on the IVIS Spectrum instrument (Perkin Elmer/Caliper Life Sciences). For EGFP imaging 465/20 nm excitation and 520/30 nm emission filters were used. For mCherry imaging, 570/20 nm excitation and 620/30 nm emission filters were used. For BphP1 imaging, 675/20 nm excitation and 720/30 nm emission filters were used. Throughout the in vivo imaging session animals were maintained under anesthesia with 1.5% vaporized isoflurane. The instrument stage was temperature-controlled (37 °C) to avoid hypothermia. For ex vivo imaging, wt-*Cre*_*vasa*_ and *BphP1-Cre*_*vasa*_ mice (both males and females, 6–10 months of age) were euthanized and freshly isolated organs (brain, kidneys, liver, ear, heart, eye) were visualized side by side. Data were analyzed using Living Image v.4.3 software (Perkin Elmer/Caliper Life Sciences). Data analysis were performed using an OriginPro v.9.7.188. For AkaLuc bioluminescence imaging (AkaBLI) in vivo, the animals were injected with NIR-emitting luciferin analog AkaLumine-HCl (Sigma-Aldrich, 808350, 75 nmol/g of body weight) 15 min before imaging. For bioluminescence detection in vitro primary cells were supplemented with 250 μM AkaLumine-HCl. Detection was performed in bioluminescence mode with an open emission filter.

### BphP1 intracellular concentration

The intracellular BphP1 concentrations were estimated based on either the PA signal or intrinsic BphP1 NIR fluorescence from specific cells in culture or tissue. For calibration of the PA and fluorescence signals, we used Eppendorf tubes with the same volume of the purified BphP1 of known concentrations ranging from 10 nM to 10 µM. To determine BphP1 concentration in 4T1 cells (ATCC), cells were pelleted down in the Eppendorf tube, adjusted to the same volume as the purified BphP1 samples and imaged side by side using the PA or fluorescence modes. The imaging data of the sample were processed using Origin software v.9.8 (OriginLab). The in vivo detection sensitivity of PAT was then estimated by dividing the BphP1 cellular concentration by the image contrast to noise ratio (*i.e*., noise equivalent detection sensitivity).

### Photoacoustic tomography in vivo

In PAT, as photons propagate in tissue, some are absorbed by biomolecules, and their energy is partially or completely converted into heat. The heat-induced pressure propagates in tissue and is detected outside the tissue by an ultrasonic transducer or transducer array to form an image that maps the original optical energy deposition in the tissue. In this study, we have developed two PAT systems for the whole-body imaging of the BphP1 transgenic mice. The PA and ultrasound imaging analysis was performed using Matlab 2019b.

### Whole-body PAT with a ring-shaped ultrasound array

The whole-body PAT system has been upgraded from our previous work^23^ (Fig. 5b). To photoswitch and image BphP1, we have combined two continuous-wave semiconductor lasers at 635 nm and 790 nm (CivilLaser) and an optical parametric oscillator laser (Surelite, Continuum) pumped by an Nd:YAG laser with a 10 Hz pulse repetition rate. The 750 nm light from the optical parametric oscillator laser is used for both whole-body PA imaging and switching off BphP1 at the same time, while the 635 nm light from the continuous-wave laser is used for switching on BphP1. The second 790 nm continuous-wave laser is used to accelerate the switching-off process. The two continuous-wave laser beams are combined by a dichroic mirror and delivered through an optical bundle with 8 output branches. The optical parametric oscillator laser beam is delivered through free space, using a conical lens (131–1290, EKSMA Optics) and a condenser (ACL7560U-B, Thorlabs) to form a ring-shaped light pattern around the animal’s trunk. The thickness of the light pattern is ~5 mm, and its diameter is similar to the cross-sectional diameter (~2−3 cm) of a mouse. The maximum light fluence on the skin of the animal is ~5 mJ/cm^2^), which is well below the American National Standards Institute (ANSI) laser safety limit. The PA signals are detected by a ring-shaped ultrasonic transducer array (Imasonic) with an 8 cm diameter, 4 MHz central frequency, more than 80% one-way bandwidth, and 512 elements. Each element is cylindrically focused to form an approximately uniform imaging region with a 40 mm diameter and 1 mm thickness. Within this region, the radial resolution is 150 µm, and the tangential resolution is 150–300 µm. The data acquisition system is adapted from a commercial Vesasonics system (Vantage 256, Verasonics) with 2-fold multiplexing. The cross-sectional imaging speed is 0.2 s per frame. A universal back-projection algorithm is used for the image reconstruction^45^.

The whole-body PACT system used in this study has been upgraded from our previous setup^23^, mainly in the imaging speed and the detection sensitivity (Supplementary Fig. 9). Our previous imaging system only had a 64-channel data acquisition system, and thus 8 laser pulses were needed to complete one image frame (i.e., 0.8 s per frame) (Supplementary Fig. 9a). The relatively slow data acquisition resulted in the overall slow imaging speed, and more importantly, the image formation process was sensitive to breathing motion artifacts of the small animals (Supplementary Fig. 9b). The motion reduced the system’s detection sensitivity of the BphP1 signals, because of the image blurring and the mismatch between the ON and OFF images (Supplementary Fig. 9c). Therefore, we previously had to acquire a large number of photoswitching cycles and average the frames to smooth out the motion artifacts, which further reduced the imaging speed. By contrast, the upgraded imaging system used in this study has a 256-channel data acquisition system, which is 4-times faster than the previous system. Each image frame takes only 0.2 s, and the impact of breathing motion on the imaging formation process is negligible (Supplementary Fig. 9d). The higher imaging speed also reduces the motion artifacts between the ON and OFF state images and thus can improve the detection sensitivity of the BphP1 signals.

### Dual-modality PA and ultrasound imaging system

The dual-modality PA and ultrasound imaging system has been adapted from a commercial small-animal ultrasound platform (Supplementary Fig. 7)^46^. For PA imaging, the hybrid system has similar photoswitching and PA imaging principles. A 635 nm continuous-wave semiconductor laser is used to switch on BphP1, while an optical parametric oscillator laser (Vscan, Spectra-Physics) at 750 nm provides the light for PA imaging and switching off BphP1 (Supplementary Fig. 8a). The two laser beams are combined by a dichroic mirror and guided into a line fiber bundle that forms a rectangular light band on the mouse’s abdominal side. The PA signals are detected by a linear ultrasonic transducer array (L7-4, Philips) with a 5 MHz central frequency, more than 50% one-way bandwidth, and 128 elements. Each element is cylindrically focused to form an approximately uniform imaging region with a focal length of 20 mm and 1 mm thickness. Within this region, the axial resolution is 300 µm, and the lateral resolution is 300–450 µm. The data acquisition system is adapted from the commercial Vesasonics system (Vantage 256, Verasonics) with no multiplexing. The cross-sectional frame rate is 10 Hz. A universal back-projection algorithm is used for the image reconstruction^45^.

For ultrasound imaging, a high-frequency dual-element focused ultrasound wobbler is used to transmit and receive ultrasound waves, with a high-frequency inner element (35 MHz) and a low-frequency outer element (2 MHz). The wobbler is made of lead zirconatetitanate (PZT) and has a diameter of 9 mm and a focal length of 19 mm. The two elements are concentrically aligned with a shared focal length. The dual-element focused transducer is used for B-mode ultrasound and acoustic angiography imaging, providing high-resolution images of mouse organ structures and vasculature perfusion at deep regions of interest. The B-mode ultrasound mode uses the inner element for both high-frequency signal transmission and receiving, and is achieved by vibrating the wobbler at a frame rate of up to 7 Hz (Supplementary Fig. 8b). The lateral resolution of the ultrasound imaging is 150 μm and the axial resolution is 50 μm. The acoustic angiography mode uses the outer element to burst microbubbles flowing in blood vessels and uses the inner element to receive super-harmonic bubble bursting signals, resulting in high-contrast vasculature images^46–48^ (Supplementary Fig. 8c). For this, 5-μm microbubbles (VesselVue, Sonovol, Inc.) were injected via the mouse tail vein right before the imaging.

The linear transducer for PA imaging and the wobbler for ultrasound imaging are mounted on the same translation motor stage, and thus the two image modalities can be accurately co-registered. An FPGA-based control box is programmed to synchronize the laser firing, motor stage motion, and PA/ultrasound data acquisition. The ultrasound and acoustic angiography signals are acquired by a single-channel high-speed data acquision (DAQ) card. The PA signals are acquired by a programmable ultrasound scanner. While the linear array provides axial cross-sectional images for the PA mode, the fast-scanning of the wobbler provides axial cross-sectional images for the ultrasound and acoustic angiography mode. The two transducers are mounted on a robotically actuated carriage with their relative positions fixed. The carriage moves along a two-dimensional motorized stage for acquiring 3D images with a large field of view. The key acoustic, optical (except the laser), scanning, and electronic components are fully immersed in a hydrocarbon fluid-filled reservoir, which improves the system’s lubrication, acoustic coupling, and heat dissipation. The reservoir has an optically and acoustically transparent imaging window on the top. The animal lies on the top of the imaging window with a natural prone position.

### Imaging processing in differential PAT

We applied a Hilbert transform to the reconstructed PA images to extract the signal envelopes, which induces some blurring to the images. Additionally, a 3 by 3 median filter was applied to the Hilbert filtered images. When the differential images (e.g., Fig. 6e) are displayed, we applied a threshold of three times the noise level, estimated as the standard deviation of the background signals outside the imaged region.

### AAV injection in mouse liver and kidney for PA imaging

Homozygous *loxP-BphP1* mice (males, 6 months of age, total 2 mice) were anesthetized with 5% isoflurane in 40% oxygen balanced with nitrogen for anesthesia induction, and then placed on a stereotaxic frame. They were inhaled with 2% isoflurane through the mask during the surgical procedure, and body temperature was maintained at 37 °C. Mice were kept at the prone position for kidney injection or the left lateral position for liver injection. The right low back area or right upper abdomen area was shaved before surgery and then cleaned with iodine and alcohol. A 1 cm longitudinal skin incision was made below the rib edge at the location of 1 cm lateral to the midline and the muscle of the abdominal wall was cut using a high-temperature cautery loop tip. Two small retractors were placed and the kidney or liver was dissected and exposed. A 25 G needle of 5 µl Hamilton syringe was inserted into the kidney or liver for 5 mm, and 1 µl of Cre-expressing AAV vector was slowly injected (AAV8 for livers and AAV2 for kidneys). The needle remained in the position for 5 min and then was removed. The muscle and skin layers were sutured separately. Isoflurane was turned off, and mice were allowed to wake up. Triple antibiotics ointment was applied on the surface of the skin incision. Post viral injection, mice were imaged at three-time points: day 19, day 23, and day 30.

### Partial hepatectomy for PA imaging

Homozygouse *BphP1-Cre*_*vasa*_ mice (males, 6 months of age, total 2 mice) were anesthetized with isoflurane in 40% oxygen balanced with nitrogen, intubated orally and ventilated mechanically. Body temperature was controlled by surface heating and cooling. The upper abdomen area was shaved and cleaned with iodine and alcohol. A 2.5 cm midline skin incision was made and the underlying muscle was cut to expose the liver. Using a cotton tip and tweezer, a 3-0 sterile surgical suture (Ethicon, R-184) was placed around the median lobe of the liver and gently tightened. The liver tissue was cut until 2 mm from the knot. The abdominal cavity was washed using sterile saline until the liquid was completely clean. The muscle and skin layers were sutured intermittently using a 6-0 nylon suture. Triple antibiotic ointment was applied to the surface of the skin incision. Then isoflurane was discontinued to allow mice to wake up and return to the home cage. Carprofen (5 mg/kg) and enrofloxacin (5 mg/kg) were subcutaneously given before surgery and 3 days after surgery. The liver region of the mice was imaged by the dual-modality PA and ultrasound imaging system before the surgery as the baseline, and then longitudinally monitored 3, 6, 9, and 13 days post-surgery to observe the liver regeneration.

### Statistics and reproducibility

No statistical method was used to predetermine sample size. No data were excluded from the analyses. Mice were randomized (simple randomization, randomization of animal sex and age) between independent experiments. Within experiments, 2 of 3 measurements were performed on littermates (same age and sex) to reduced the influence of other possible variables. The investigators were not blinded to allocation during experiments and outcome assessment. Data on PCR genotyping were always reproducible in our routine screening for *n* = 86 *loxP-BphP1* mice, *n* = 80 *BphP1-Cre*_*vasa*_ mice (Fig. 1c) and for *n* = 3 independent brain samples (Supplementary Fig. 3d). All mouse genotypes identified with PCR were reconfirmed by in vivo fluorescence imaging (as in Fig. 1e, h). Fluorescence imaging of wild-type, hemizygous and homozygous *BphP1-Cre*_*vasa*_ pups (Fig. 3c) shows typical data of *n* = 7 independent experiments, reconfirmed with subsequent tissue PCR genotyping in 3 independent primary cell culture isolations. Fluorescence images following Cre recombination in vitro (Fig. 1f, g) show typical data reproduced in 3 independent experiments on hippocampal neurons and 3 independent experiments on fibroblasts. Fluorescence imaging of wt-*Cre*_*vasa*_ and *BphP1-Cre*_*vasa*_ mouse organs (Fig. 2) shows typical data reproduced in 2 independent experiments. Non-invasive PA imaging of BphP1-expressing embryo in vivo (Fig. 6) shows typical data reproduced in 1 independent experiment with 7 individual embyros. For other experiments *n* is provided in the Figure legends.

### Reporting summary

Further information on research design is available in the Nature Research Reporting Summary linked to this article.

## Supplementary information

Supplementary Information

Reporting Summary

## Data Availability

All data supporting the findings of this study are available within the article, Supplementary Information and Source Data file. The major plasmids constructed in this study will be deposited to Addgene (#186186, #186187). The *loxP-BphP1* mice will be donated to the Jackson Laboratory Repository (JAX #036061). Source data are provided with this paper.

## Source data

Source Data File

## Code availability

The image processing code used in this study are available at Duke Photoacousic Imaging Lab GitLab page https://gitlab.oit.duke.edu/pilab/optogenetic_nir_pat
