# Supplementary material for: Optogenetic manipulation and photoacoustic imaging using a near-infrared transgenic mouse model
Source: Nat Commun. 2022 May 19;13:2813. doi: 10.1038/s41467-022-30547-6 (PMC9120076; doi:10.1038/s41467-022-30547-6)
Supplement: Supplementary file 1 — Supplementary Information [file 41467_2022_30547_MOESM1_ESM.pdf]

Optogenetic manipulation and photoacoustic imaging using a near-infrared transgenic mouse model

Supplemental Information

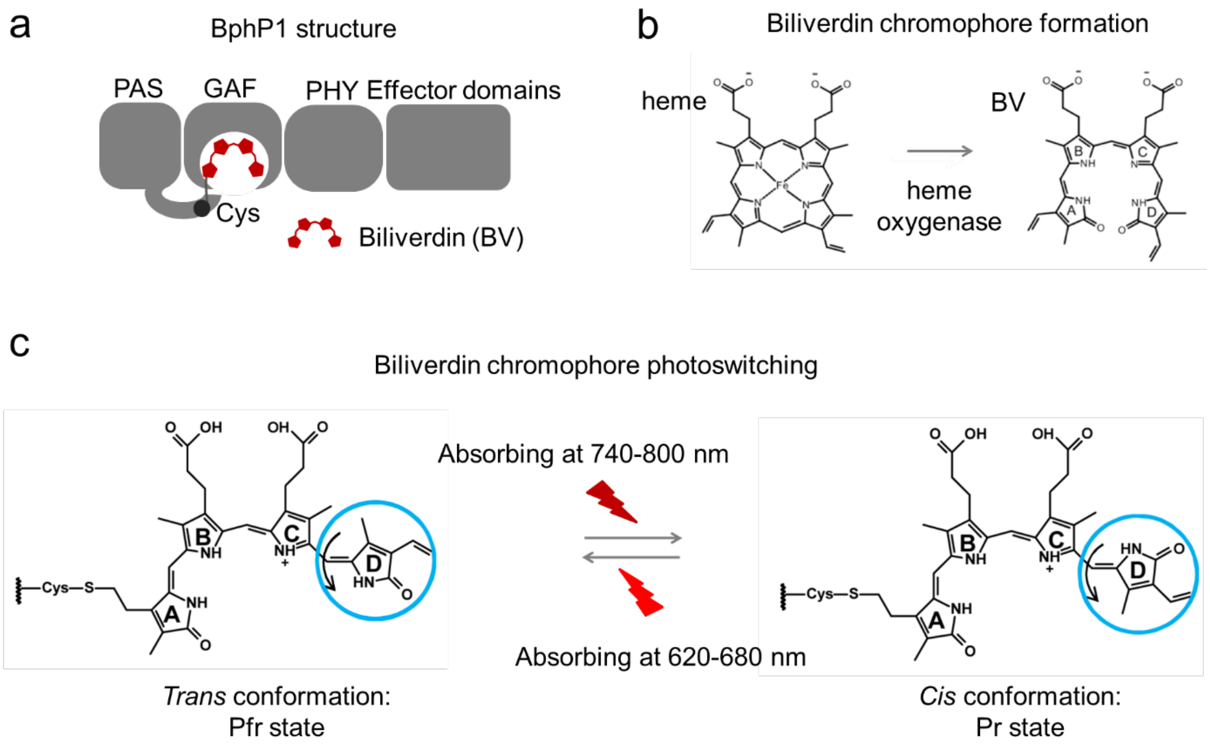

Supplementary Figure 1. Structure and photochemical properties of bacterial phytochrome photoreceptor BphP1. (**a**) Organization of a monomer subunit of BphP1. (**b**) Enzymatic synthesis of biliverdin IXα (BV) from a heme. (**c**) Photoswitching of a BV chromophore from the Pfr state to the Pr state, and vice versa, induced by NIR (~740-800 nm) light and far-red (~620-680 nm) light illumination, respectively. The photoswitching result from the out-of-plane rotation (black arrows) of the D-ring of BV about the adjacent C15/16 double bond between the C and D pyrrole rings.

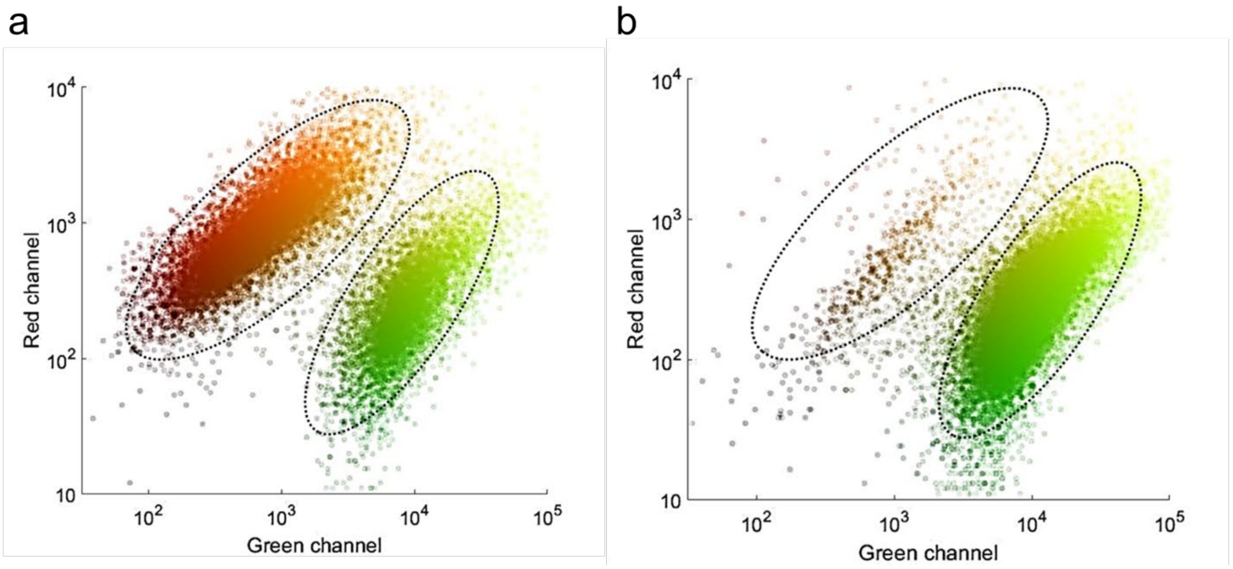

Supplementary Figure 2. Flow cytometry analysis of Cre-recombination in primary skin fibroblasts. Cells were isolated from *loxP-BphP1* mouse and transduced with AAV6-Cre (multiplicity of infection 10^5) (**a**) or left untreated (**b**). Seven days after transduction, cells were detached with trypsin, resuspended in an ice-cold cell sorting buffer (PBS with 2% FBS and 5 mM EDTA) and analyzed on the LSRII flow cytometer. For (a, b), the initial single-cell gating was performed using forward and side light scattering (FSC-A / SSC-A). These cells were then used to access fluorescence in green (for EGFP) and red (for mCherry) channels. Gates for quantification of EGFP versus mCherry fluorescence brightness are shown. For the green channel, a 488 nm laser, a 525/50 nm excitation filter, and a 505 nm DLP dichroic were used. For the red channel, a 561 nm laser, a 610/20 nm excitation filter, and a 600 nm DLP dichroic were used.

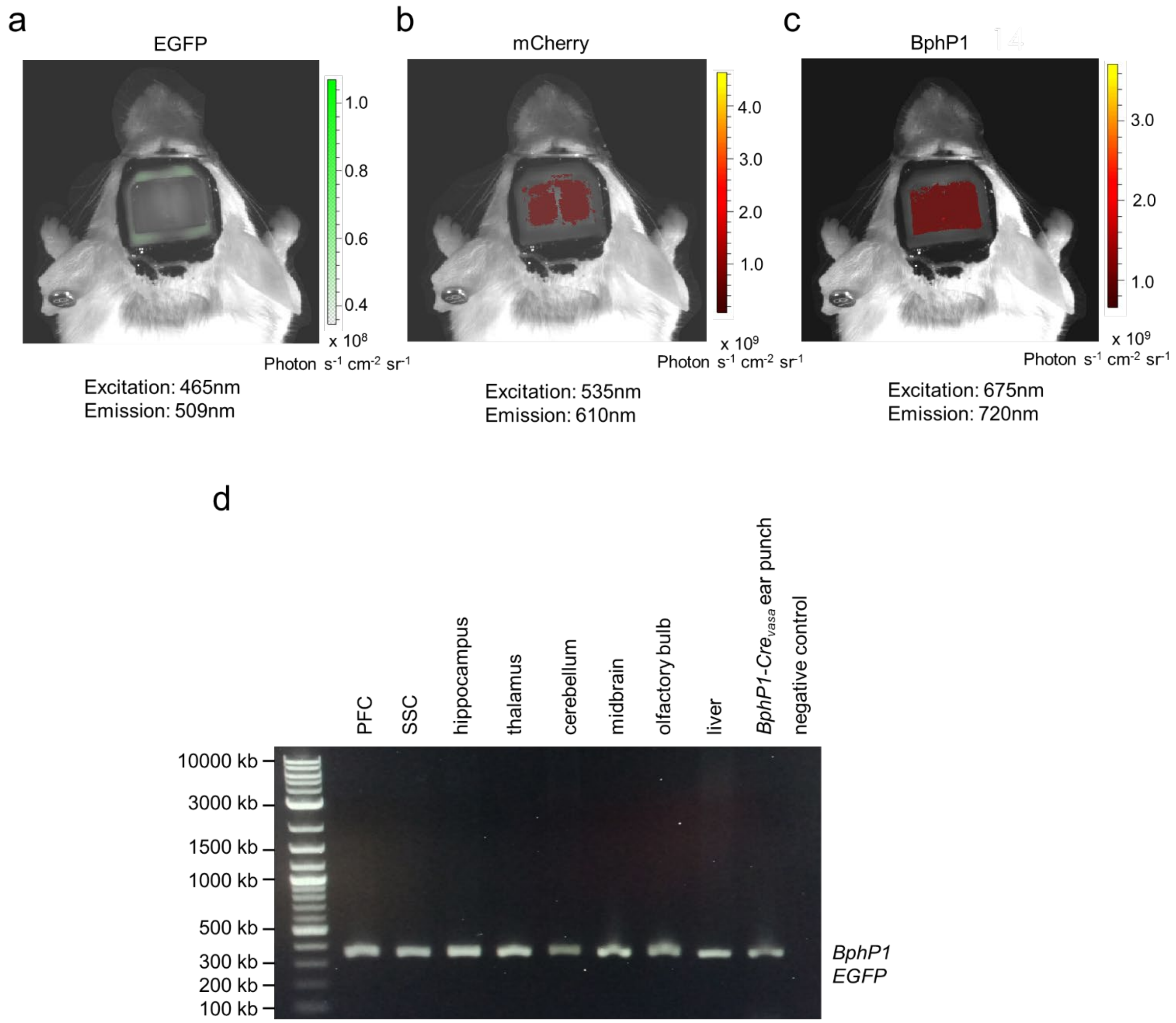

Supplementary Figure 3. Fluorescence imaging and PCR analysis of *BphP1-Cre$_{vasa}$* mouse brain. (a-c) Cranial window, showing **(a)** no EGFP signals, **(b)** strong mCherry signals, and **(c)** strong BphP1 signals acquired by IVIS Spectrum. (**d**) PCR analysis of individual brain regions and the liver of the *BphP1-Cre$_{vasa}$* mouse. Uncut agarose gel with PCR amplification products is shown. PFC, prefrontal cortex; SSC, somatosensory cortex. For more details, see the "Statistics and reproducibility" section of the Methods.

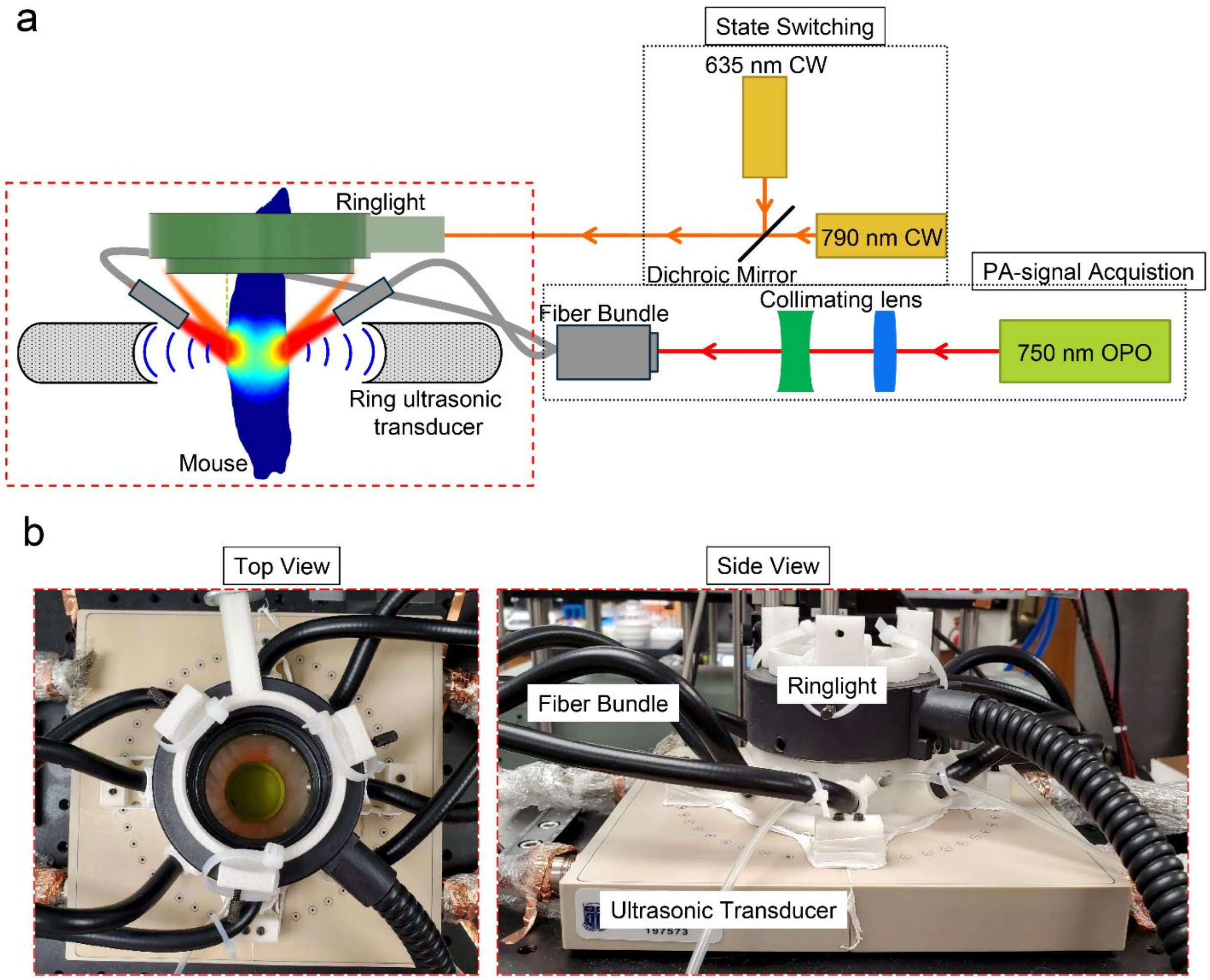

Supplementary Figure 4. Ring-array-based photoacoustic imaging system. (a) Schematic of the imaging system, showing the combination of photoacoustic excitation light at 750 nm and the photoswitching light at 635 nm and 790 nm. CW, continuous-wave semiconductor laser; OPO, optical parametric oscillator laser. **(b)** Photos of the system from the top view and side view.

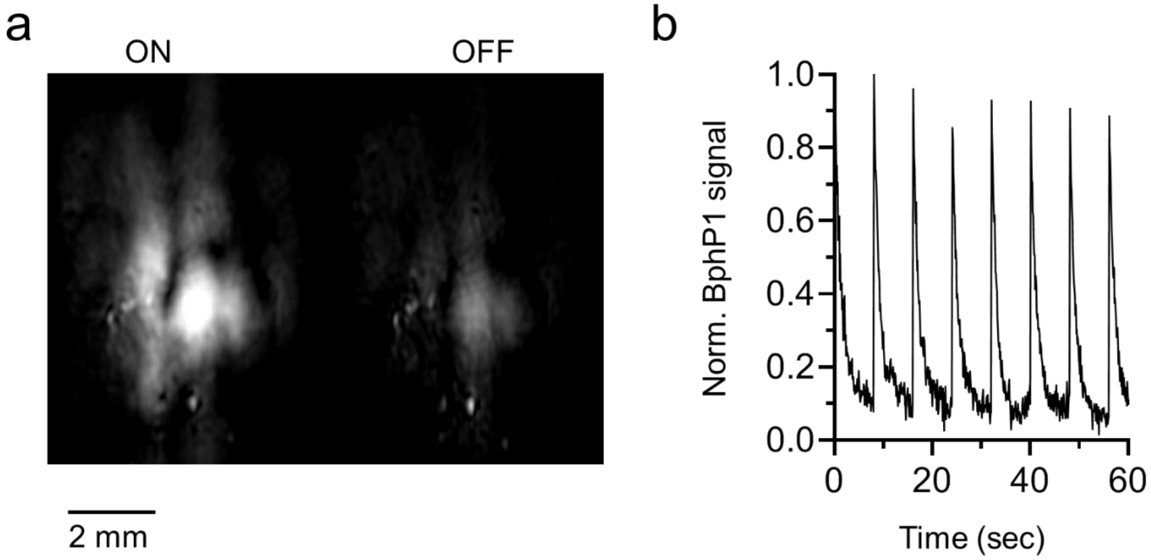

Supplementary Figure 5. Photoswitching and photoacoustic imaging of BphP1-expressing 4T1 cells. (a) The PA images of the 4T1 cells embedded in agar at ON (Pfr) and OFF (Pf) states. **(b)** The time course of the PA signals of the 4T1 cells in repeated photoswitching.

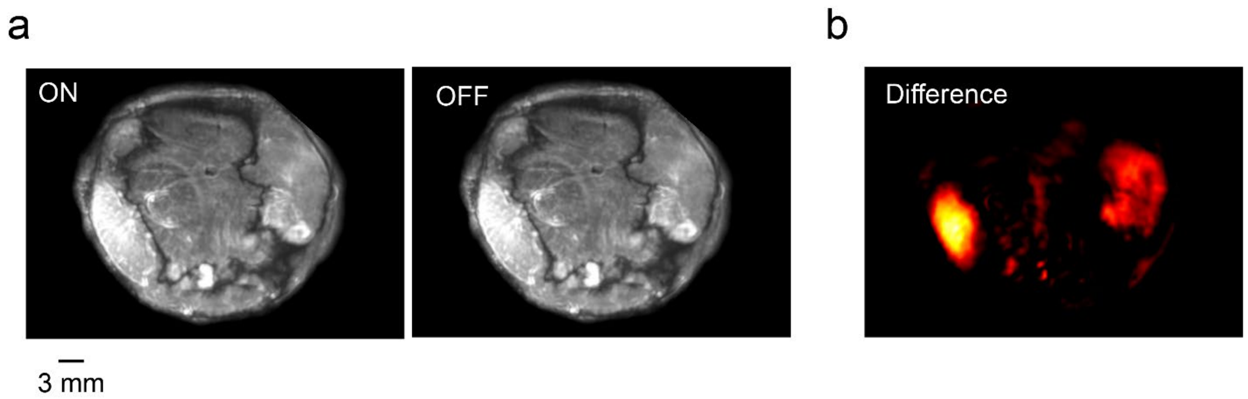

Supplementary Figure 6. Photoswitching and photoacoustic imaging of *BphP1-Cre$_{vasa}$* mouse. **(a)** Representative images of the whole-body PA images of the *BphP1-Cre$_{vasa}$* mouse, with the BphP1 in ON and OFF states, showing the dominant signals from hemoglobin. **(b)** The differential image shows the BphP1 signals with the hemoglobin signals suppressed.

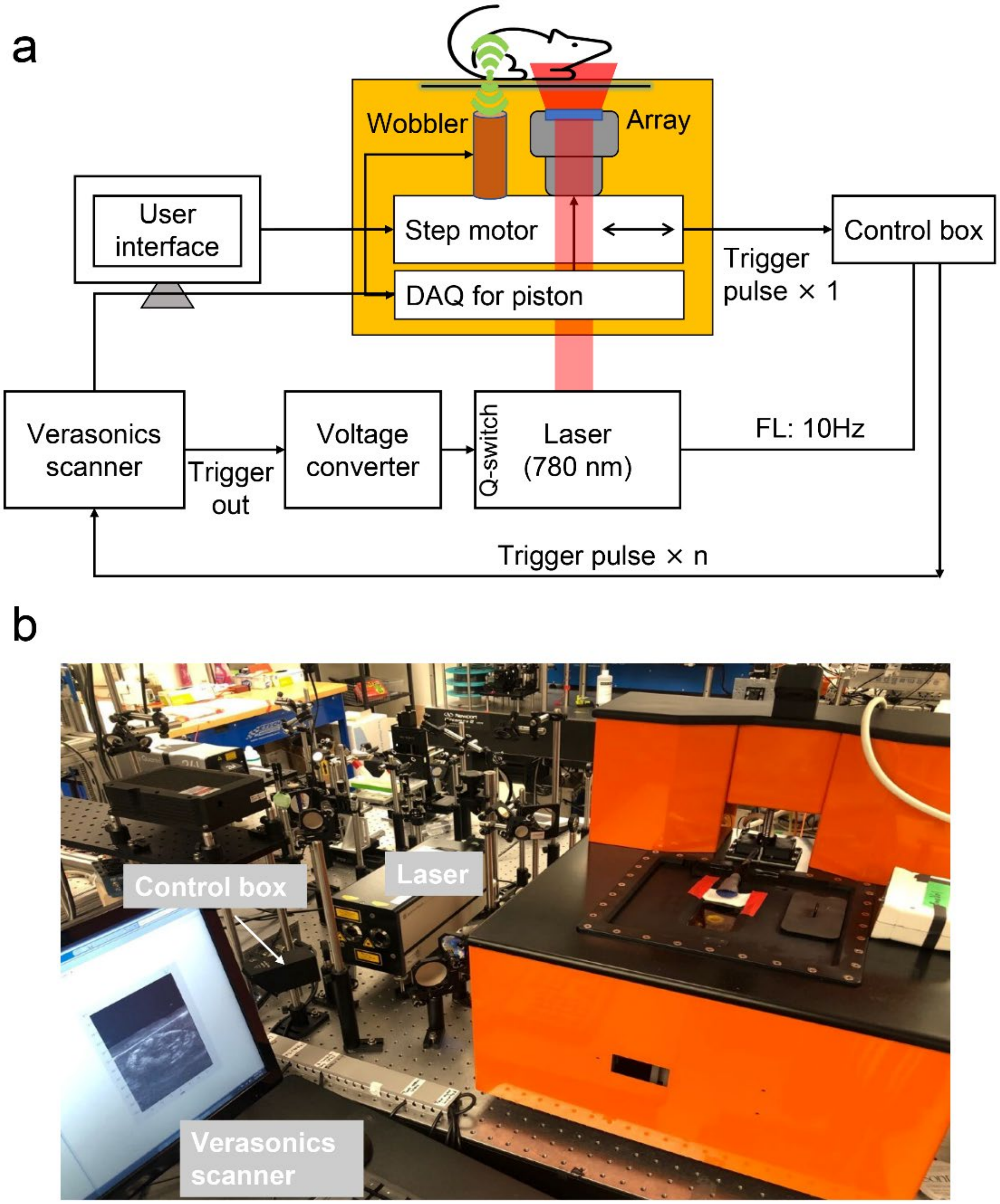

Supplementary Figure 7. Dual-modality photoacoustic and ultrasound imaging system. (a) Schematic of the imaging system, showing the arrangement of the linear ultrasound transducer for photoacoustic imaging, and the focused wobbler transducer for ultrasound and acoustic angiography imaging. DAQ, data acquisition. **(b)** Photo of the imaging system.

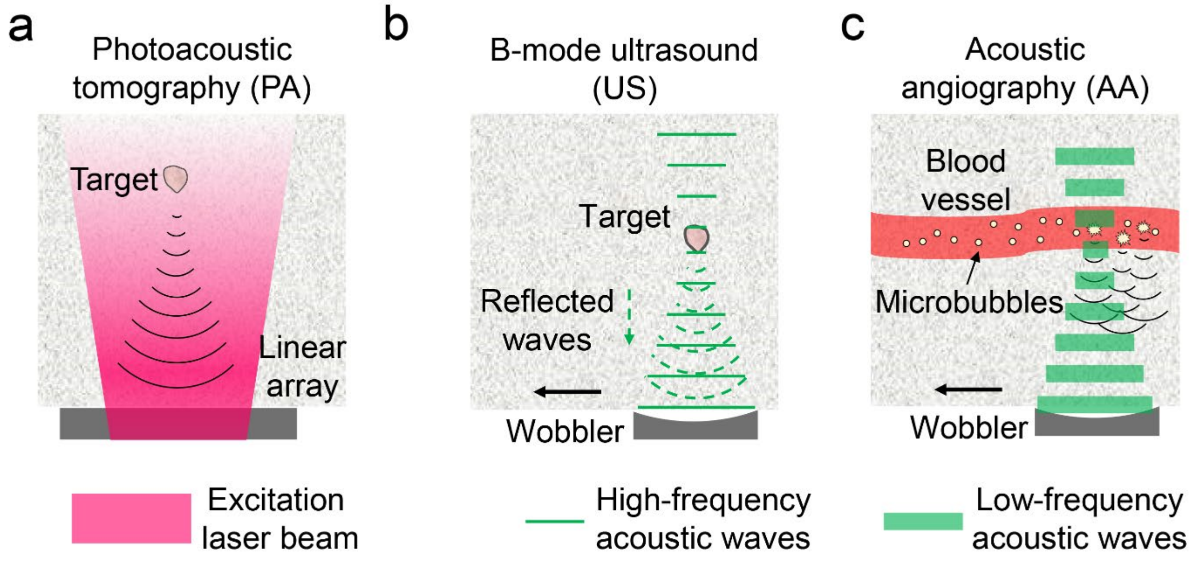

Supplementary Figure 8. Imaging principles of the dual-modality photoacoustic and ultrasound imaging system. **(a)** Photoacoustic imaging uses pulsed laser light for excitation and a linear transducer array for signal detection. **(b)** B-mode ultrasound imaging transmits and receives the high-frequency ultrasound waves using the focused wobbler transducer. **(c)** Acoustic angiography detects the harmonic signals from gas-filled bubbles as the exogenous contrast agent to image the blood vessels.

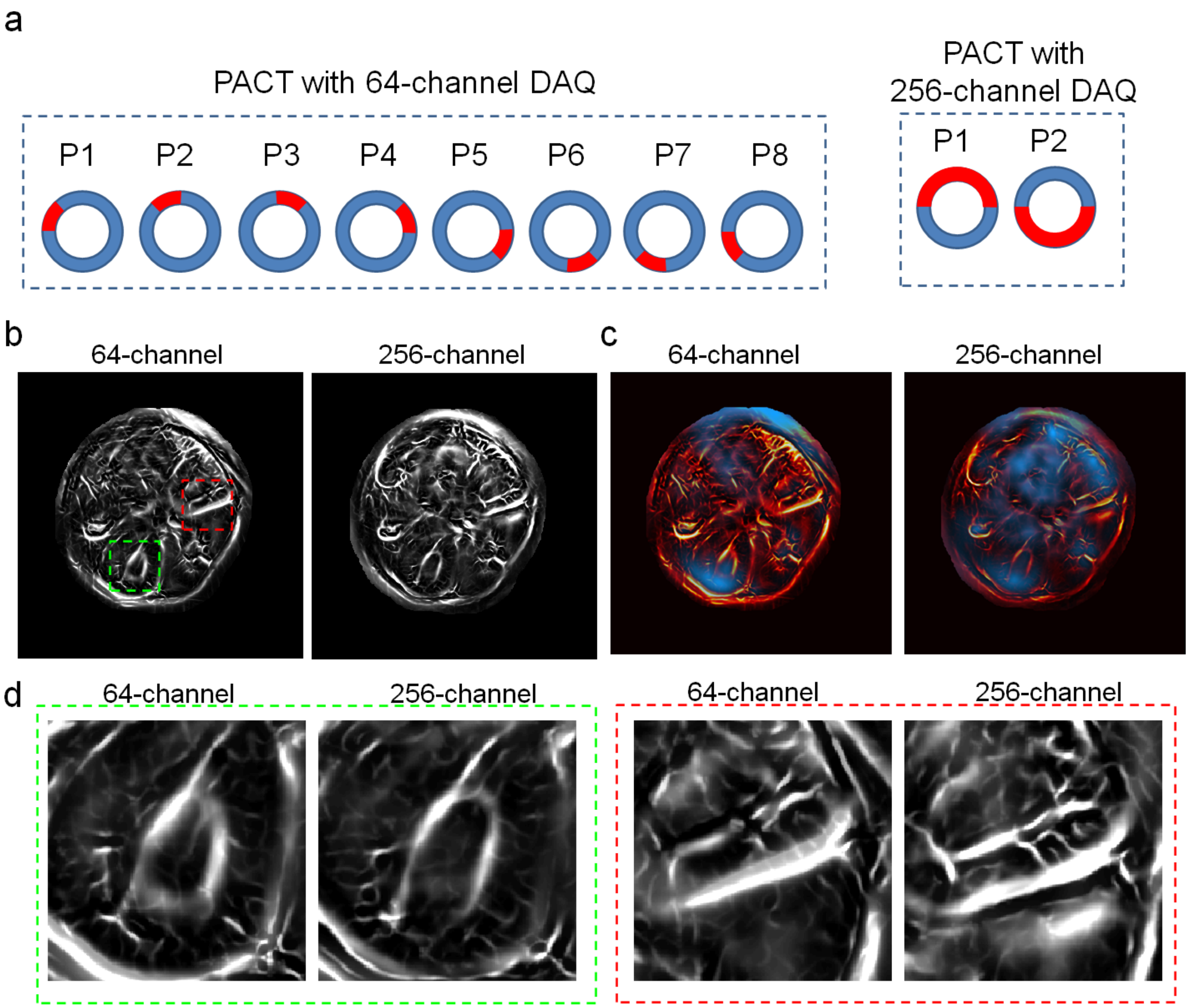

Supplementary Figure 9. Comparison of imaging performance of PACT with 64-channel or 256-channel data acquisition (DAQ). (a) Data acquisition scheme of 64-channel PACT and 256-channel PACT. While the 64-channel system needs 8 laser pulses to form one image, the 256-channel system only needs 2 laser pulses and thus is 4 times faster. **(b)** *BphP1-Cre*$_{vasa}$ mouse cross-sections images of the kidney region as acquired by a 64-channel system and 256-channel system. **(c)** The photoswitching BphP1 signals (shown in blue) as acquired by the 64-channel and 256-channel systems, clearly show that the 256-channel system can better detect the BphP1 signals with improved sensitivity. **(d)** Close-up images of the green and red boxes in **(b)**, showing better reconstruction accuracy and reduced motion-induced blurring by the 256-channel system.
