# Supplementary material for: Optogenetic manipulation and photoacoustic imaging using a near-infrared transgenic mouse model
Source: Nat Commun. 2022 May 19;13:2813. doi: 10.1038/s41467-022-30547-6 (PMC9120076; doi:10.1038/s41467-022-30547-6)
Supplement: Supplementary file 2 — Reporting Summary [file 41467_2022_30547_MOESM2_ESM.pdf]

nature portfolio

Corresponding author(s): Junjie Yao, Vladislav Verkhusha

Last updated by author(s): May 2, 2021

Reporting Summary

Nature Portfolio wishes to improve the reproducibility of the work that we publish. This form provides structure for consistency and transparency in reporting. For further information on Nature Portfolio policies, see our Editorial Policies and the Editorial Policy Checklist.

Statistics

For all statistical analyses, confirm that the following items are present in the figure legend, table legend, main text, or Methods section.

n/a	Confirmed	
[ ]	[x]	The exact sample size (n) for each experimental group/condition, given as a discrete number and unit of measurement
[ ]	[x]	A statement on whether measurements were taken from distinct samples or whether the same sample was measured repeatedly
[x]	[ ]	The statistical test(s) used AND whether they are one- or two-sided<br>*Only common tests should be described solely by name; describe more complex techniques in the Methods section.*
[x]	[ ]	A description of all covariates tested
[x]	[ ]	A description of any assumptions or corrections, such as tests of normality and adjustment for multiple comparisons
[ ]	[x]	A full description of the statistical parameters including central tendency (e.g. means) or other basic estimates (e.g. regression coefficient) AND variation (e.g. standard deviation) or associated estimates of uncertainty (e.g. confidence intervals)
[x]	[ ]	For null hypothesis testing, the test statistic (e.g. F, t, r) with confidence intervals, effect sizes, degrees of freedom and P value noted<br>*Give P values as exact values whenever suitable.*
[x]	[ ]	For Bayesian analysis, information on the choice of priors and Markov chain Monte Carlo settings
[x]	[ ]	For hierarchical and complex designs, identification of the appropriate level for tests and full reporting of outcomes
[x]	[ ]	Estimates of effect sizes (e.g. Cohen's d, Pearson's r), indicating how they were calculated

Our web collection on statistics for biologists contains articles on many of the points above.

Software and code

Policy information about availability of computer code

Data collection	The Olympus IX81 microscope was operated with a SlideBook v.6.0.8 software (Intelligent Imaging Innovations) or µManager 1.3 (Vale Lab, UCSF). Flow cytometry data were collected using BD LSRII flow cytometer and BD FACSDiva v.8.0.1 (BD Biosciences) software. In vivo fluorescence and bioluminescence imaging data collected with IVIS Spectrum instrument (PerkinElmer/Caliper Life Sciences). The PA and ultrasound imaging were collected using the imaging systems developed in the Junjie Yao laboratory at Duke University.
Data analysis	IVIS Spectrum data were processed using a Living Image v.4.3 software (Perkin Elmer/Caliper Life Sciences). Data plotting and analysis were performed using OriginPro v.9.7.188 or OriginPro v.9.8 software (OriginLab). The PA and ultrasound imaging analysis was performed using Matlab2019b. The image processing code used in this study are available at Duke Photoacousic Imaging Lab GitLab page https://gitlab.oit.duke.edu/pilab/optogenetic_nir_pat.

For manuscripts utilizing custom algorithms or software that are central to the research but not yet described in published literature, software must be made available to editors and reviewers. We strongly encourage code deposition in a community repository (e.g. GitHub). See the Nature Portfolio guidelines for submitting code & software for further information.

Data

Policy information about availability of data

All manuscripts must include a data availability statement. This statement should provide the following information, where applicable:

- Accession codes, unique identifiers, or web links for publicly available datasets
- A description of any restrictions on data availability
- For clinical datasets or third party data, please ensure that the statement adheres to our policy

All data supporting the findings of this study are available within the article and Supplementary Information/Source Data file. All other information related to the

findings of the study are available from the corresponding authors upon reasonable request for research use. The major plasmids constructed in this research, their maps and sequences are deposited to Addgene depository. The loxP-BphP1 mice will be donated to the Jackson Laboratory Repository (JAX #036061).

Field-specific reporting

Please select the one below that is the best fit for your research. If you are not sure, read the appropriate sections before making your selection.

- [x] Life sciences
- [ ] Behavioural & social sciences
- [ ] Ecological, evolutionary & environmental sciences

For a reference copy of the document with all sections, see nature.com/documents/nr-reporting-summary-flat.pdf

Life sciences study design

All studies must disclose on these points even when the disclosure is negative.

Sample size	Sample size comprises n=3 independent experiments, e.g., 3 mice per each of 3 tested groups (total 9 mice), or 3 independent isolations of primary cells of each type (neurons, fibroblasts and endothelial cells).<br>Several-fold difference between groups and low variability of the data allow to rely on this sample size and confer to the requirements of the Association for Assessment and Accreditation of Laboratory Animal Care (AAALAC) International standards for the humane care and use of animals (https://www.aaalac.org/the-guide/), in particular, the minimization of the number of animals used to achieve specific scientific objectives.
Data exclusions	No data were excluded.
Replication	All attempts at replication were successful. The number of independent experiments (biological replicates) is indicated in Figure legends or/and "Statistics and reproducibility" section.
Randomization	For primary cell cultures, simple randomization was performed before transduction with AAVs. Cells were isolated from both male and female mouse pups.<br>According to ARRIVE (Animal Research Reporting of In Vivo Experiments) guidelines, mice were randomized (simple randomization, randomization of animal sex and age) between independent experiments. Within experiments, 2 of 3 measurements were performed on littermates (similar age and sex) to reduced the influence of other possible variables as recommended in https://onlinelibrary.wiley.com/doi/10.1002/eji.201142048.
Blinding	The investigators were not blinded due to the nature of performed optogenetic and PAT experiments.

Reporting for specific materials, systems and methods

We require information from authors about some types of materials, experimental systems and methods used in many studies. Here, indicate whether each material, system or method listed is relevant to your study. If you are not sure if a list item applies to your research, read the appropriate section before selecting a response.

Materials & experimental systems

n/a	Involved in the study	
x		Antibodies
	x	Eukaryotic cell lines
x		Palaeontology and archaeology
	x	Animals and other organisms
x		Human research participants
x		Clinical data
x		Dual use research of concern

Methods

n/a	Involved in the study	
x		ChIP-seq
	x	Flow cytometry
x		MRI-based neuroimaging

Eukaryotic cell lines

Policy information about cell lines

Cell line source(s)	4T1 tumor cells were obtained from ATCC. AAV-293T cells were from Agilent.
Authentication	Cell lines were not additionally authenticated.
Mycoplasma contamination	Cell lines were not additionally tested for mycoplasma.
Commonly misidentified lines (See ICLAC register)	No commonly misidentified cell lines were used.

Animals and other organisms

Policy information about studies involving animals; ARRIVE guidelines recommended for reporting animal research

Laboratory animals	The Balb/c and Balb/c x FVB F1 and F2 hybrids, newborn pups and 2- to 10-month-old mice (both males and females) from established colonies (originated from Ingenious Targeting Laboratory), with body weights of 22–32 g were used. The pregnant female mice (FVB-Tg(Ddx4-cre)1Dcas/J, 3 months old, 20-30 g body weight) were used for photoacoustic imaging.<br>Mice were maintained on a 12-h light and dark cycle at room temperature and standard humidity (~40-55%), with ad libitum access to food and water. Same-sex littermates were housed together in cages with chopped corn cob bedding (2-5 mice per cage). Environmental enrichment included pieces of compressed cotton nestles and paper huts.<br>Primary cultures of neurons, fibroblasts and endothelial cells were isolated from homozygous BphP1-Crevasa mice (both males and females).
Wild animals	No wild animals were used in the study.
Field-collected samples	No field collected samples were used in the study.
Ethics oversight	Our study complies with all relevant ethical regulations. All procedures with animals were performed in an AAALAC-approved facility and received ethical approval from the Institutional Animal Care and Use Committee of the Albert Einstein College of Medicine (protocol 00001050) and Duke University (protocol A009-20-01).

Note that full information on the approval of the study protocol must also be provided in the manuscript.

Flow Cytometry

Plots

Confirm that:

- [x] The axis labels state the marker and fluorochrome used (e.g. CD4-FITC).
- [x] The axis scales are clearly visible. Include numbers along axes only for bottom left plot of group (a 'group' is an analysis of identical markers).
- [x] All plots are contour plots with outliers or pseudocolor plots.
- [x] A numerical value for number of cells or percentage (with statistics) is provided.

Methodology

Sample preparation	Primary skin fibroblasts grown in a 24-well plate were detached with trypsin (10 min at 37oC) and resuspended in an ice-cold cell sorting buffer (PBS with 2% FBS and 5 mM EDTA) at 500,000 cells per ml.
Instrument	BD LSRII flow cytometer.
Software	BD FACSDiva v.8.0.1 (BD Biosciences)
Cell population abundance	Cell population abundance was estimated for 2 distinct populations: EGFP or mCherry positive cells. According to flow cytometry analysis, about 65% of skin fibroblasts underwent excision of loxP-flanked EGFP (Supplementary Fig. 2).
Gating strategy	Initial gates - FSC-A/SSC-A to discriminate cells from debris. Resulted population were analyzed for EGFP and mCherry fluorescence. For EGFP fluorescence measurements a 488 nm laser and a 525/40 emission filter were used. For mCherry fluorescence measurements a 561 nm laser and a 610/20 emission filter were used (Supplementary Fig. 2).

- [x] Tick this box to confirm that a figure exemplifying the gating strategy is provided in the Supplementary Information.
